# Artificial intelligence in functional food innovation: Bioactive enhancement and formulation optimization: A quasi-systematic review

**DOI:** 10.1016/j.fochx.2026.103628

**Published:** 2026-02-07

**Authors:** Nadia Alkalbani, Leen Shahin, Hiba Benzeghiba, Reyad S. Obaid, Tareq M. Osaili, Leila Cheik Ismail, Ghayah Al qasssimi, Maha Rauf, Khawla Abdulrahim, Afra Almashgouni, Fatima Ashuweihi, Dana AL-Fuqaha

**Affiliations:** a Department of Clinical Nutrition and Dietetics, College of Health Sciences, University of Sharjah, Sharjah, PO Box 27272, United Arab Emirates; b Research Institute of Medical & Health Sciences, University of Sharjah, Sharjah, PO. Box 27272, United Arab Emirates; c Department of Nutrition and Food Technology, Faculty of Agriculture, Jordan University of Science and Technology, PO Box 3030, Irbid 22110, Jordan; d Nuffield Department of Women's & Reproductive Health, University of Oxford, Oxford OX1 2JD, UK

**Keywords:** Bioactive compounds, Bioavailability, Explainable AI, Functional foods, Optimization, Metabolomics, Multi-omics

## Abstract

Artificial intelligence (AI) is increasingly integrated into functional food research. This quasi-systematic review analyzes 53 peer-reviewed studies (2015–2025) to outline current applications and emerging directions, including the underexplored domain of antioxidant food development. The review attempts to provide an updated synthesis of AI approaches across compound discovery, metabolomics, and consumer modeling, emphasizing knowledge gaps and opportunities for methodological integration. Data-driven AI (classical machine learning) and deep learning methods have been applied to predict antioxidant activity, identify bioactive compounds, and reveal patterns in metabolomic data. Unsupervised approaches have assisted in clustering complex datasets, whereas optimization algorithms supported the adjustment of sensory, nutritional, and functional attributes. However, many current systems remain limited to *in silico* findings, lacking experimental or clinical validation. Consumer modeling remains largely predictive, with limited integration of ethical and regulatory dimensions. Continued collaboration between food scientists and data scientists is essential for translating computational insights into practical applications.

## Highlights

• A quasi-systematic review of 53 studies on AI in functional food development.
• Explainable AI ensures transparency in bioactive compound prediction.
• Deep learning and omics integration enhance functional food precision.
• Convolutional neural networks excel in analyzing complex metabolomic data.
• Microbiota variability challenges AI-based personalized nutrition models.

## 1 Introduction

Recently, functional foods have become a focus in food science as they provide physiological benefits and contribute to the prevention of noncommunicable diseases (Topolska et al., 2021). These products may occur naturally or be obtained through processing and are often enriched with bioactive compounds, such as vitamins, polyphenols, prebiotics, and fatty acids (Banwo et al., 2021; Khan et al., 2013). The inclusion of these components extends health benefits beyond basic nutrition, prompting a shift from conventional composition analysis toward the deliberate design of bioactive-rich foods with targeted physiological functions (Sgroi et al., 2024). Additionally, the functional food sector has experienced rapid innovation and market expansion, with diverse formulations introduced continuously. In 2018, the global functional food market was valued at approximately USD 170 billion and is projected to approach USD 300 billion by 2025, with growth led primarily by Asia, followed by the USA, Canada, and Europe (Capanoglu et al., 2024; Sgroi et al., 2024).

The increasing demand for functional foods has been accompanied by efforts to develop innovative production strategies. The development of novel functional products requires prior identification and classification of bioactive compounds, followed by the evaluation of their behavior within food matrices and assessment of their stability (Ahmad & Al-Shabib, 2020; Banwo et al., 2021). Equally important is the understanding of the molecular-level mechanisms through which these compounds exert their health-promoting effects (Ahmad & Al-Shabib, 2020). Alongside these technical considerations, ethical aspects play a critical role in ensuring responsible development and long-term sustainability. Transparency regarding applied approaches and their implications is particularly important as neglecting ethical considerations may undermine consumer trust, create regulatory challenges, and hinder adoption of functional food innovations (Yang, Jiao, et al., 2025).

Recently, artificial intelligence (AI) has become an essential facilitator in functional food research, providing analytical tools across multiple subfields. Specifically, AI-driven frameworks in the food sector commonly categorize approaches into rule-based systems, data-driven AI based on classical machine learning (ML), and deep learning (DL), offering a structured basis for diverse applications. These frameworks support advanced analysis in food chemistry and contribute to addressing emerging challenges, such as four-dimensional food structures and intelligent bio-nanocomposite packaging (Ekrami et al., 2025; C. Gu et al., 2026; Mobahi et al., 2025). Additionally, AI tools enable dynamic forecasting of food quality, sensory attributes, and flavor chemistry (Sung et al., 2025; Yang, Zhu, et al., 2025).

Within this landscape, classical ML methods are widely applied for predictive modeling and pattern recognition in complex datasets, supporting compound evaluation and consumer stratification (Bennett-Lenane et al., 2022; Dakal et al., 2024). Supervised algorithms rely on labeled data to predict defined outcomes, such as bioactivity scores or sensory ratings. In contrast, unsupervised models identify latent structures in unlabeled datasets and are therefore well suited for exploratory tasks, including metabolomic clustering and ingredient profiling (Dickinson et al., 2018). Meanwhile, DL approaches, particularly convolutional and recurrent neural networks, provide enhanced capabilities for analyzing spectral, imaging, and omics data characterized by high dimensionality and nonlinear relationships (Martorell-Marugan et al., 2019; Wang, Tian, et al., 2024). These models enable automatic feature extraction and often outperform traditional methods in applications, such as bioactivity prediction and formulation optimization **(****Jiang et al.,** 2025**;** **Thakur et al.,** 2023**).** Complementarily, natural language processing (NLP) facilitates the extraction of insights from unstructured sources, including scientific literature, regulatory documents, and consumer reviews (Canatan et al., 2025; Chhetri, 2023; Mezgec et al., 2019).

In addition to individual applications, AI-integrated databases and computational platforms are being explored as tools for mapping and classifying food components and examining their links to physiological effects (Flores Martinez et al., 2024**;** Meima et al., 2023**).** In general, these tools form an integrated framework that accelerates discovery, supports precise formulation, and aligns functional food development with health priorities, regulatory expectations, and evolving market demands.

Recent reviews have provided valuable insights into AI-assisted discovery of food-derived bioactive compounds, particularly peptides, with emphasis on model development, screening strategies, and high-throughput prediction (Chang et al., 2025; Doherty et al., 2021; Y. Zhang et al., 2024). Notably, while AI-based approaches for bioactive compound discovery and identification have been extensively reviewed, their integration into the development of antioxidant-rich foods remains limited. Therefore, the present review updates current knowledge while extending the scope beyond early-stage compound identification.

Accordingly, this review aims to: (1) investigate AI-assisted bioactive component analysis; (2) explore AI applications in formulation and process optimization; (3) discuss strategies for enhancing compound stability and bioavailability; (4) evaluate the role of AI in decoding market trends and consumer behavior; and (5) examine the limitations and ethical implications of AI integration, including modeling of the human gut microbiota and considerations related to personalized nutrition. This structure is designed to provide a coherent synthesis that balances technical depth with practical relevance.

**Fig. 1:**
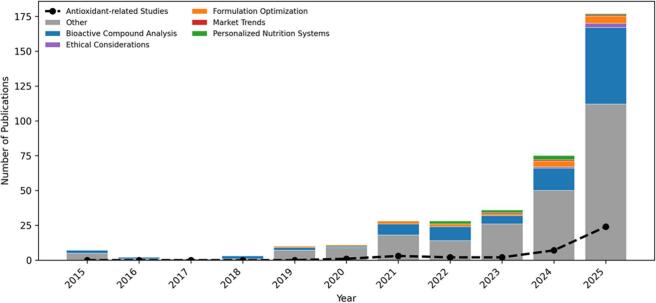
Bibliometric overview of AI applications in functional food research (2015–2025), based on records retrieved from Scopus, showing annual publication trends across major research domains. The dashed line highlights the temporal growth of AI-driven antioxidant-related studies. AI: artificial intelligence.

Fig. 1 presents a bibliometric overview (2015–2025) illustrating publication trends and their distribution across the five thematic domains addressed in this review. Among the 362 publications identified during this period, 39 explicitly focus on antioxidant, polyphenol, or phenolic properties (black dashed line), representing a subset within the broader domain of bioactive compound analysis.

By integrating AI-based modeling with biological insight and regulatory considerations, this review delineates the current landscape of AI in functional food development, identifies critical gaps, and outlines directions for ethical and data-driven innovation.

## 2 Methodology

This quasi-systematic review provides a structured synthesis of the literature at the intersection of AI and functional food development. Relevant publications were retrieved from Scopus, Web of Science, and PubMed using search terms, such as ‘functional foods,’ ‘artificial intelligence in food science,’ ‘machine learning for the prediction of bioactive compounds,’ and related phrases. The searches were limited to peer-reviewed articles published between 2015 and 2025. The inclusion criteria were: (1) peer-reviewed articles published between 2015 and 2025; (2) original research articles, clinical trials, and review papers; and (3) studies addressing AI-driven approaches in compound profiling, food formulation, personalized nutrition, and consumer behavior modeling. The exclusion criteria were: (1) preprints; (2) conference proceedings; and (3) AI applications unrelated to food science. The selected literature was thematically classified into five analytical domains to ensure complete coverage and reflect methodological diversity: bioactive compound analysis, formulation optimization, personalized nutrition systems, market trends, and ethical considerations. Data management and descriptive summaries were performed using Microsoft Excel, while Plotly in Python was used for trend visualization. All abbreviations used in this manuscript are defined in the Supplementary Tables A.1 and A.2.

## 3 AI applications in bioactive compound analysis

AI-based approaches are increasingly used to accelerate the identification, characterization, and functional interpretation of food-derived bioactive components. This section outlines key applications across three areas: (1) The discovery and structural analysis of bioactive components driven by AI, (2) the integration of ML and DL in metabolomic data processing, and (3) the use of specialist databases to map compound bioactivity in diverse health contexts. These three areas are envisioned to form a coherent framework that advances precision, efficiency, and mechanistic understanding in the development of next-generation functional foods.

### 3.1 AI in bioactive compound discovery and characterization

ML has become an effective approach for predicting the activity of bioactive compounds in complex food matrices. These compounds, such as polyphenols, peptides, alkaloids, and polysaccharides, exert physiological functions beyond basic nutrition, such as antioxidant, anti-inflammatory, and metabolic regulatory effects (Astley & Finglas, 2016; Y. Zhang et al., 2024).

**Fig. 2:**
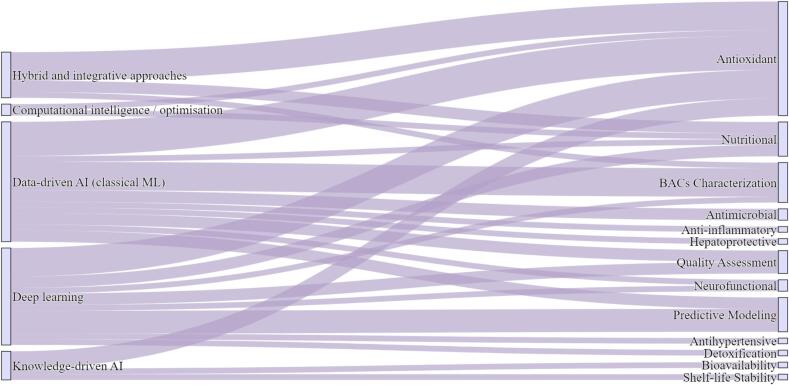
Distribution of AI approaches and their application domains in functional food research, based on selected studies published between 2015 and 2025 (*n* = 53). AI: artificial intelligence, ML: machine learning, BACs: bioactive compounds.

Fig. 2 *presents the distribution of AI approaches and their respective application areas in functional food research, based on 53 selected studies published between 2015 and 2025.* Table A.1 and Fig. 2 show that classical ML approaches dominate AI-assisted functional food studies (*n* = 21), indicating a non-random methodological preference. ML predominance is most evident in studies targeting antioxidant activity (*n* = 6) and bioactive compound characterization (*n* = 5). This focus reflects the availability of standardized *in vitro* assays, including 2,2-diphenyl-1-picrylhydrazyl (DPPH), 2,2′-azinobis (3-ethylbenzothiazoline-6-sulfonic acid) (ABTS), and ferric reducing antioxidant power, which generate consistent datasets suitable for structure-activity relationship (SAR) modeling (Alov et al., 2015; Guardado Yordi et al., 2019; W. Li et al., 2025). Methodologically, Fig. 2 further suggests that ML adoption is driven by throughput considerations. Conventional chromatographic and bioassay-based workflows are robust but operationally time- and resource-intensive, whereas ML enables rapid screening and prioritization of candidate bioactives from large datasets **(****Bennett-Lenane et al.,** 2022**;** **Park et al.,** 2022**).**

Notably, Table A.1 indicates that recent studies increasingly integrate spectral and metabolomic data rather than relying solely on molecular descriptors. Applications involving hyperspectral imaging of cocoa beans (*Theobroma cacao*) and berries (*Lycium barbarum*) as well as untargeted metabolomics for pork spoilage prediction, illustrate a methodological shift toward data-rich inputs capable of capturing complex food matrices (Caporaso et al., 2018; M. Gu et al., 2023; Hu et al., 2024).

**Fig. 3:**
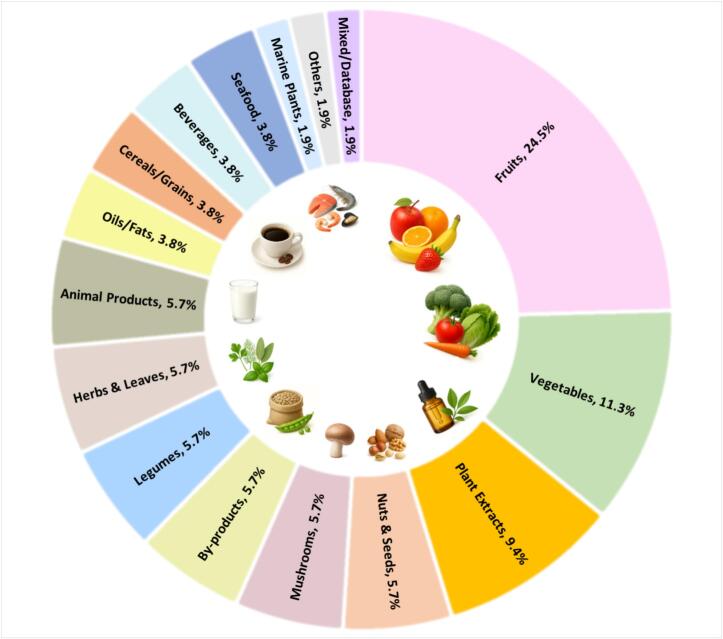
Distribution of food matrices employed in **selected** AI-assisted functional food studies published from 2015 to 2025 *(n = 53)*. AI: artificial intelligence.

Fig. 3 shows the distribution of food matrices in AI-assisted functional food studies from 2015 to 2025 (*n* = 53). Fruits, vegetables, and plant extracts constitute the majority of the investigated matrices, consistent with the dominance of antioxidant-focused modeling approaches. In contrast, cereals and marine plants are underrepresented (≤3.77%), which may reflect analytical complexity and the limited availability of standardized datasets for these matrices.

Overall, we suggest that these patterns point to opportunities for future AI-assisted functional food research. Expanding ML applications toward anti-inflammatory, antimicrobial, neuroprotective, and anticancer endpoints may be supported by harmonized bioassays, improved data annotation, and stronger integration between food science and pharmacology.

Supervised algorithms, such as Random Forest (RF), Support Vector Machines (SVM), and Partial Least Squares Discriminant Analysis (PLS-DA), have been widely applied in SAR modeling of food-derived compounds (Table A.1) (Caporaso et al., 2018; Y. Liu et al., 2023; Rico et al., 2023; Sanchez-Reinoso et al., 2025). More recently, DL models, such as bidirectional long-short-term memory (Bi-LSTM), have shown improved performance in capturing structural and physicochemical patterns. For example, W. Li et al. (2025) applied a Bi-LSTM model trained with fusion features to predict the antioxidant potential of food-derived peptides. The accuracy improvement was mainly because of the Bi-LSTM architecture, which captured long-range dependencies in peptide sequences. In contrast, the integration of quantum chemical descriptors did not raise predictive accuracy. Instead, it enabled mechanistic interpretation by linking electronic properties, such as the Highest Occupied Molecular Orbital-Lowest Unoccupied Molecular Orbital gap, with antioxidant potential.

Furthermore, unsupervised learning methods are increasingly used in functional food research to reveal underlying patterns in metabolomic data, as summarized in Table A.1 (Dickinson et al., 2018; M. Gu et al., 2023; Squara et al., 2024). Several studies have implicitly applied phenotypic clustering approaches, through the grouping of samples based on biochemical profiles. These applications have been reported in contexts, such as stress response in legumes (*Medicago truncatula)* by Dickinson et al. (2018), quality classification in hazelnuts)*Corylus avellana* L. (by Squara et al. (2024), and spoilage prediction in pork (*Sus scrofa domesticu*) by M. Gu et al. (2023). These findings can enrich existing food compound libraries (*e.g.*, FooDB, Phenol-Explorer, and PhytoHub (with novel insights into metabolomic patterns and phenotypic traits. For example, K-means clustering was used to classify 1069 mutant lines of colored wheat based on seed color parameters (L*, a*, and b*), with the resulting clusters associated with antioxidant activity and agronomic traits (Hong et al., 2025). In another study, time-resolved metabolomic analysis under abiotic and biotic stress conditions revealed metabolic shifts involving compounds, such as sucrose, citric acid, and tetrahydroxychalcone, which emerged as potential indicators of stress-responsive functionality (Dickinson et al., 2018).

Based on these pattern-recognition techniques, recent efforts have focused on improving model interpretability. One approach involves using methods that attribute biological outcomes to specific input variables. For instance, in a study by Zhao et al. (2025), SHAP was applied in conjunction with RF regression to evaluate changes in antioxidant activity during apricot kernel fermentation with *Lactiplantibacillus plantarum*. The model identified hydroxybenzoic acid, L-cystine, and L-anserine as key contributors to improved antioxidant responses. This integration of predictive modeling with interpretative tools supports a deeper understanding of how compositional changes during fermentation influence functional outcomes.

In a related application, the GutBug model was developed using ML classifiers equipped with interpretability tools to predict microbial enzymes responsible for metabolizing dietary components, such as flavonoids and prebiotics. The model achieved predictive precisions between 78% and 97% by mapping enzyme-substrate interactions across enzyme commission classes and validating results against known biochemical reactions (Malwe et al., 2023). This approach enhances the identification of gut bacteria and their enzymes, facilitating interpretation of metabolite-microbe interactions critical to microbiome-driven functional food development.

Additionally, recent efforts have focused on combining DL with advanced sensing techniques to enhance analytical precision. For example, Y. Liu et al. (2023) used terahertz time domain spectroscopy integrated with a convolutional neural network (CNN) to classify dried tangerine peel (*Pericarpium Citri Reticulatae*) by storage duration, outperforming traditional chemometric methods (PLS-DA, RF, and LS-SVM). Similarly, a multitask 1D CNN with an attention mechanism was used by Hu et al. (2024) to predict the content of polyphenols and polysaccharides in goji berries from hyperspectral images. The 1D CNN, well-suited for sequential spectral data, facilitated efficient learning from the high-dimensional inputs and significantly enhanced prediction accuracy. Notably, while these models predict compositional patterns, they do not directly establish functional activity. Functional inference requires subsequent biological validation through *in vitro*, *in vivo*, or clinical studies to confirm physiological relevance. Symbolic AI represents another pathway, offering rule-based systems that enhance model transparency and are particularly effective in data-scarce environments (Magnini et al., 2023). These models have been applied by Ganje et al. (2019) to simulate compound release kinetics and structural behavior in amylose-encapsulated orange peel oil, providing mechanistic insights where empirical data are limited.

In summary, AI-driven discovery in this field has progressed from basic predictive modeling to DL architectures and explainable AI (XAI) frameworks, which aim to improve both accuracy and biological interpretability. Rule-based systems complement these predictions by integrating domain-specific knowledge, particularly in data-scarce environments. However, significant challenges remain: the heterogeneous nature of food matrices complicates model generalizability, while issues of overfitting and class imbalance (which can bias models against rare bioactive patterns) persist (Gygi et al., 2023; Periwal et al., 2022). Addressing these gaps requires expanding high-quality datasets and ensuring rigorous experimental validation to confirm functional efficacy, thereby ensuring that predictive accuracy is not conflated with true biological understanding.

### 3.2 AI for metabolomics and high-throughput data analysis

Metabolomic data sets are highly complex, often involving thousands of metabolites in diverse environments under various experimental conditions. This dimensionality challenges conventional statistical approaches, which may reveal only partial biological patterns. These include principal component analysis (PCA), linear discriminant analysis (LDA), hierarchical cluster analysis (HCA), and PLS-DA. However, AI-based methods, especially supervised algorithms, such as SVM, RF, and neural networks, facilitate the identification of discriminative metabolic signatures linked to health outcomes or specific functional food interventions (Koraqi et al., 2025; Qian et al., 2024; Rong et al., 2024). One example is the use of a RF screening model to reveal SARs in sleep-promoting peptides derived from casein hydrolysates. Through the integration of peptidomics and ML, bioactive peptide candidates were identified and subsequently supported by *in silico* and *in vivo* validation (Qian et al., 2024). Complementing these supervised approaches, unsupervised learning techniques also have potential in exploring underlying patterns in high-dimensional data. For instance, Kohonen self-organizing maps, an unsupervised DL method, have been applied by Koraqi et al. (2025) to classify Kosovan honey types based on their physicochemical and antioxidant profiles. This approach facilitated dimensionality reduction and clear clustering of samples according to botanical origin, revealing subtle yet meaningful variations in bioactive content.

Functional foods exert their effects through multilevel interactions between dietary components and biological systems (Jones & Jew, 2007). Consequently, metabolomics alone may capture only partial molecular responses, particularly in untargeted workflows lacking predefined hypotheses (Chi et al., 2024). To overcome these limitations, hybrid frameworks integrating conventional statistical approaches with AI are increasingly adopted. Such integrations reduce noise, optimize computational efficiency, and prioritize biologically relevant features (Breda et al., 2024; Squara et al., 2024). Particularly, ML models facilitate the integration of heterogeneous untargeted datasets, thereby enabling the identification of cross-layer regulatory mechanisms and novel bioactive targets that remain inaccessible to conventional methods. Z. Wang et al. (2023) exemplify this approach, in which untargeted metabolomics based on ultra-performance liquid chromatography coupled with quadrupole time-of-flight mass spectrometry was used. The resulting data were analyzed using ML algorithms, including SVM, RF, and back propagation neural networks (BPNN), to distinguish *Prunus pseudocerasus* and *Prunus tomentosa*, two closely related Chinese cherry species. The BPNN achieved the highest classification accuracy, identifying flavonoids, such as procyanidin B1 and (*epi*)catechin, as key contributors to antioxidant differences (Z. Wang et al., 2023).

Although PCA and HCA remain useful for clustering and dimensionality reduction, they are limited to linear, descriptive patterns and lack predictive capability. In contrast, AI-based models capture nonlinear relationships, integrate diverse data types, and generate quantitative predictions, thereby extending analytical scope (Breda et al., 2024; Squara et al., 2024; Z. Wang et al., 2023). Based on this, graph convolutional networks support mechanistic inference across omics layers by modeling biochemical interactions, as demonstrated by **Gonzalez et al. (**2021**)** in predicting anticancer bioactive compounds**. As summarized in Table A.1,** multitask DL models further enhance efficiency by profiling multiple bioactive components simultaneously, for example through hyperspectral imaging in plant-based matrices, such as goji berries (*Lycium barbarum*) **(****Hu et al.,** 2024**).**

Compared to DL architectures, classical ML models typically depend on manual feature extraction, which constrains their ability to capture complex biological variability. In contrast, DL architectures, such as CNNs and recurrent neural networks, learn hierarchical representations directly from high-dimensional omics datasets, enabling more effective modeling of nonlinear and interdependent relationships (Martorell-Marugan et al., 2019). This capability is particularly relevant in metabolomics, where variable interactions rarely follow linear patterns. Within this context, CNNs have demonstrated strong performance in analyzing two-dimensional correlation spectroscopy (2D-COS) data derived from metabolomic fingerprints. For instance, Dong et al. (2022) transformed near-infrared hyperspectral data into 2D-COS images and applied CNN-based classification to determine the geographic origin of wolfberry samples. The CNN models outperformed conventional classifiers, such as LDA and SVM, achieving higher accuracy and robustness. This example illustrates how DL enhances feature extraction from spectral data and strengthens the reliability of origin verification and quality control in food systems.

Furthermore, hybrid architectures, such as Bi-LSTM-CNN, models have shown potential in peptide classification tasks by integrating both sequential and contextual features of amino acid chains. In this setting, CNN layers capture local structural motifs, while Bi-LSTM components model longer-range semantic relationships across sequences (Y. Li et al., 2022). From an analytical perspective, such architectures may reduce reliance on manual preprocessing by enabling end-to-end learning, in which raw peptide sequences are mapped directly to predictive outputs. This characteristic may be particularly relevant for multifunctional peptides, where conventional models often encounter difficulty in resolving overlapping structural and functional signals.

Additionally, AI-driven approaches have advanced metabolite deconvolution and biological pathway inference, supporting a more mechanistic interpretation of compound behavior in functional food systems (Gao et al., 2022). Wang, Holscher, et al. (2025) developed a DL model (McMLP) to predict metabolite-level responses to dietary interventions by jointly modeling dietary inputs, microbiome shifts, and downstream metabolites, such as short-chain fatty acids (SCFAs) and bile acids. The resulting framework revealed pathway-level interactions that are difficult to resolve using traditional analytical pipelines. Similarly, Wang, Tian, et al. (2024) addressed spectral overlap in wine metabolomics by integrating 2D-COS with CNN-based classification, achieving over 96% accuracy in geographic origin tracing. *These findings support the view presented in this review; domain-specific AI can enhance compound classification, highlight correlations, and prioritize candidate compounds relevant to biological pathways in food systems.*

*Overall, AI-driven metabolomics has evolved from traditional statistical tools to a complex landscape of supervised and unsupervised models, alongside DL architectures and hybrid frameworks (e.g., Bi-LSTM-CNN and AI-statistical combinations) that optimize feature extraction and biochemical mapping. Despite this progress, significant gaps remain, including limited model transparency* (Salahuddin et al., 2022), data heterogeneity, batch effects, and a scarcity of annotated datasets. Consequently, future research should integrate attention mechanisms to enhance interpretability (Wang, Ta, et al., 2024) and use model distillation to develop computationally efficient, lightweight frameworks (G. Yang et al., 2023). These advancements are crucial for transitioning from predictive profiling to a validated, mechanistic understanding of bioactive-metabolite interactions in functional foods.

### 3.3 AI for bioactivity mapping through specialized databases

Unlike static repositories, such as PubChem, contemporary platforms, such as FooDB, NutriChem, and the Human Metabolome Database (HMDB) integrate chemical structures, metabolic pathways, and *in vitro*/*in vivo* bioactivity profiles within interconnected frameworks. These databases facilitate AI-driven compound discovery by enabling pattern recognition across diverse data types. ML algorithms, particularly NLP and graph-based models, reveal relationships between food-derived metabolites and health outcomes that remain undetected through conventional approaches (Amara et al., 2022; Chi et al., 2024).

Table 1 illustrates the role of specialized bioactivity databases in linking molecular-level data with AI-based predictive models. It presents 11 distinct and complementary databases. As a whole, these databases support AI-driven functional food innovation and differ in focus, ranging from metabolomic profiling to nutrient composition and flavor modeling. Database integration supports multi-level analysis. HMDB compiles chemical, clinical, and nutritional data, enabling researchers to link metabolic signatures with functional properties. As listed in Table 1, Ayyash et al. (2022) demonstrated this by profiling fermented date fruit pomace, associating specific metabolites with antioxidant and antidiabetic effects. Similarly, combining FooDB, Phenol-Explorer, and NEVO databases allows compound-level dietary assessment, shifting focus from broad nutrient classes to individual bioactive contributions (Meima et al., 2023).

**Table 1:** Overview of the selected food-related databases, their key focus areas, and representative applications in AI-driven functional food research.

Database	Focus Area	Application in AI	Reference
PubChem BioAssay	Compound-target interactions and experimental bioassay data	Model training for activity prediction, toxicity assessment, virtual screening	(Zhang, Ding, et al., 2025)
ChEMBL	Bioactive molecules with drug-like properties and target annotations	Machine learning classifier training for bioactivity prediction, mechanism elucidation	(Westerman et al., 2020)
FooDB	Chemical composition of food items	Ingredient similarity analysis, clustering, food-chemical profiling	(Meima et al., 2023)
Phenol-Explorer	Polyphenol content in foods	Discovery of bioactive compounds, antioxidant modeling	(Meima et al., 2023)
PhytoHub	Phytochemicals in edible plants	Plant-based compound tracking, bioactivity fingerprinting	(Scalbert et al., 2014)
USDA FoodData Central	Nutrient composition of foods (macro and micronutrients)	Ingredient profiling, dietary intake analysis, nutrition modeling	(Delgado et al., 2021)
COCONUT	Natural products and phytochemical structures	Deep learning-based bioactivity prediction, compound similarity modeling	(Zhang, Ding, et al., 2025)
BindingDB	Protein-ligand binding affinity data (Kd, Ki, and IC₅₀)	Network-based bioactivity mapping, protein target identification	(Lacroix et al., 2018)
NEVO	National food composition database (Dutch foods)	Country-specific AI models, regional nutrient analysis	(Meima et al., 2023)
FlavorDB	Flavor molecules and sensory properties	Flavor prediction, sensory-based recommendation, taste mapping	(Garg et al., 2017)
HMDB	Human metabolome (including food-related metabolites)	Metabolomic pattern detection, health-outcome prediction, food-disease interaction	(Ayyash et al., 2022)

AI: artificial intelligence; IC₅₀: half-maximal inhibitory concentration; Ki: inhibition constant; Kd: dissociation constant.

AI integration transforms these repositories into predictive tools. As shown in Table 1, Lacroix et al. (2018) constructed polyphenol-protein interaction networks using BindingDB and pathway databases, identifying potential targets for dietary polyphenols. Building on this foundation, Westerman et al. (2020) developed *PhyteByte*, a ML classifier trained on ChEMBL data to predict bioactivity of food compounds in FooDB. More recently, Zhang, Ding, et al. (2025) applied DL through Chemprop to predict antibacterial activity, training on PubChem BioAssay data and extending predictions to the COCONUT natural products database.

In addition to public resources, commercial platforms integrate AI with proprietary libraries. Brightseed® applied computational prioritization to identify N-trans-caffeoyltyramine and N-trans-feruloyltyramine in hemp hulls, compounds subsequently validated for enhancing microbiome diversity and SCFA production (Flores Martinez et al., 2024). This shift from empirical screening to prediction-guided design demonstrates practical applications of AI-database integration.

Network-based visualizations and pathway enrichment tools enhance interpretability, bridging computational predictions with biochemical mechanisms. As these platforms advance, they transition from reference repositories to discovery engines, supporting mechanistic insights in functional food development.

## 4 AI in the optimization and formulation of functional foods

Integrating AI into functional food formulation offers a structured method for solving complex multivariate design problems. This section highlights how selected AI techniques, including multi-objective optimization (MOO), genetic algorithms (GAs), fuzzy logic (FL), and heuristic search methods, are applied to balance nutritional value, sensory quality, cost, and safety. Table 2 displays the selected studies conducted to optimize and formulate functional foods and related products.

**Table 2:** Overview of selected studies (2015–2025) exploring AI-driven optimization and formulation strategies in functional food and related systems.

Food /Process	AI Model / Algorithm	Objective	Key Findings	Reference
Amaranth and oat-based gluten-free pasta	FL	Optimize extrusion parameters and flour ratio for sensory acceptability	Determined optimal screw speed, moisture, temperature, and flour mix for best sensory outcomes	(Sakre et al., 2016)
Ultrasound hydration process for finger millet	ANN + PSO	Reduce antinutrients (phytates and tannins) and enhance functional properties	Achieved a 73% reduction in phytate and 71% tannin reduction, improved absorption and digestibility	(Dubey & Tripathy, 2024)
Black carrot juice processing	Gradient Boosting + FL	Optimize thermosonication for bioactive retention and sensory quality	Identified conditions that improved bioactive and sensory ratings through a combined GB and fuzzy evaluation	(Yikmis et al., 2025)
Coconut milk spray-drying	PSO + ANN	Predict the quality of coconut milk powder from spray drying	Outperformed basic ANN; Inlet temperature was the most critical factor	(Ming et al., 2021)
Low-sodium fish sauce (via electrodialysis)	PSO	Optimize salt reduction in fish sauce while maintaining nitrogen and aroma compound content	The optimal salt content predicted by PSO aligned with the results of independent sensory test results	(Ratanasanya et al., 2018)
Mixed foods and raw agri. Products	Multilayer Perceptron	Predict antiradical potential across diverse food types	Predicted antioxidant capacity for 1315 items.	(Gorbachev et al., 2022)
Animal (swine) diet formulation	Bayesian Optimization	Optimize digestible energy, lysine content, and cost simultaneously	Outperformed previous stochastic methods, improving objectives by∼10%, 14%, and 3%, respectively	(Uribe-Guerra et al., 2024)
Curcumin-loaded liposome formulation	Ensemble models	Optimize entrapment efficiency under different formulation conditions	Determined optimal formulation conditions	(Hoseini et al., 2023)
Oil-in-water emulsion + orange by-product flour ± soy protein	ANN	Stability of the model of emulsions with orange by-product	Predicted emulsion stability under varying conditions	(Umaña et al., 2022)
Blended frying oil formulation	GA	Generate optimal oil blends that balance frying stability, nutritional value (lower saturated fats), and cost	Achieved oil blends with frying stability similar to palm oil but with reduced saturated fat and lower cost, validated through heating and frying experiments	(Liang et al., 2022)
Root phenotypes of maize and common bean	MOEA	Optimize nutrient uptake, biomass, and carbon cost	Identified diverse phenotypes with trade-offs between growth, uptake, and root carbon efficiency	(Rangarajan et al., 2022)
Mixed fruit beverage (amla, pineapple, and coconut water)	FL	Optimize blend composition based on sensory data	Identified optimal mix and supported precise thermal treatment, ensuring quality and microbial safety	(Dhar et al., 2021)
Soy protein-based food foam	FL	Optimize foaming capacity and stability via process parameters	Achieved optimal SPI, sonication, and whipping conditions; whipping time had the greatest impact on foam quality.	(Güldane, 2023)
Multigrain beverage premix (barley, millet, quinoa, and yellow split pea)	MOGA + Fuzzy analysis	Optimize ingredient ratios for energy value and cost	Identified optimal blend; results confirmed by fuzzy analysis	(Thakur et al., 2023)
Enzyme-ultrasound-assisted extraction of mulberry anthocyanins	DNN	Optimize solid-liquid ratio, ethanol concentration, ultrasonic temperature, and pectinase	Dosage to maximize a weighted CE index (TAC, C3G, and C3R)	(Zhang, Jiang, et al., 2025)
Mosambi (Citrus limetta) peel – de-bittering process	VM-ANN-GA and SVM-GPR-GA	Optimize NaCl concentration and soaking time to maximize polyphenols and antioxidant activity with acceptable sensory quality	Achieved R2 > 0.99 for predictive accuracy; GA optimization identified ∼10% NaCl and ∼ 8 h soaking as optimal for balancing polyphenol retention (∼0.77%) and antioxidant activity (∼60%) with good sensory acceptability	(Younis et al., 2019)
Cocoa beans - non-destructive quality assessment using hyperspectral imaging	PLSR and PCR models	Predict fermentation index, total polyphenol content, and antioxidant activity from hyperspectral data of single cocoa beans	Achieved reliable prediction performance linking spectral information with biochemical quality attributes, demonstrating the potential of hyperspectral imaging for rapid quality evaluation	(Caporaso et al., 2018)

ANN: Artificial neural network, FL: Fuzzy logic, GA: Genetic Algorithm, MOGA: Multi-Objective Genetic Algorithm, MOEA: Multi-Objective Evolutionary Algorithm, PSO: Particle Swarm Optimization, DNN: Deep Neural Network, VM: Support Vector Machine, SVM: Support Vector Machine, GPR: Gaussian Process Regression, PLSR: Partial Least Squares Regression, PCR: Principal Component Regression, SPI: Soy Protein Isolate, TAC: Total Anthocyanin Content, C3G: Cyanidin-3-O-glucoside, C3R: Cyanidin-3-O-rutinoside, CE index: a Comprehensive Evaluation index derived using the entropy weight method, combining the weighted contributions of TAC, C3G, and C3R.

### 4.1 Multi-target optimization

The MOO framework evaluates multiple formulation goals simultaneously, such as nutrient density, product acceptability, cost-efficiency, and regulatory safety (Liang et al., 2022; Ming et al., 2021; Sakre et al., 2016; Yikmis et al., 2025). Unlike linear models, MOO accommodates the complexity of food systems, providing more context-sensitive solutions. It also supports formulation targeting specific population needs (Canatan et al., 2025; Wan et al., 2022; Wang, Huang, et al., 2025).

Nutritional design is inherently multidimensional. It involves enhancing bioactive components, such as fiber, antioxidants, and protein, while limiting elements, such as sodium, saturated fat, and antinutrients (Table 2) (Dubey & Tripathy, 2024; Gorbachev et al., 2022; Güldane, 2023; Liang et al., 2022; Ratanasanya et al., 2018; Thakur et al., 2023). Achievement of optimal energy density and maintenance of sensory properties, such as taste, texture, and aroma, are also key considerations (Bashiri et al., 2025; Sakre et al., 2016; Yikmis et al., 2025).

A practical example comes from Thakur et al. (2023), who used a multi-objective genetic algorithm to formulate a multigrain beverage premix. The blend included barley roasted and malted flour, pearl millet, quinoa, and yellow split pea. Particularly, roasting contributed to a notable increase in carbohydrate content. PCA and Pearson's correlation revealed strong associations between energy, carbohydrate, and fat content. FL was then used to validate the formulation, which delivered 345.8 kcal/100 g at a cost-effective rate. This approach demonstrates how an algorithm-driven formulation can integrate nutritional goals with affordability, an essential requirement for the development of accessible functional foods.

Optimization techniques in functional food formulation are based on diverse data inputs. These include nutrient profiles from food composition databases (*e.g.*, USDA FoodData Central, and NEVO), ingredient libraries (*e.g.*, FlavorDB and Phenol-Explorer) and consumer preference patterns, as elaborated in Section 6.1. These datasets provide the quantitative and qualitative foundation needed to design products that meet both health criteria and user acceptability (Abejon et al., 2020; Delgado et al., 2021).

Although MOO offers a structured approach to balancing nutritional and sensory goals, its output depends heavily on the quality of the input data and assumptions defined in its objective functions (Ferraz de Arruda et al., 2023; Sahin & Aytekin-Sahin, 2024). Overparameterization or narrow health targets can lead to results that are mathematically optimal but biologically or culturally unsuitable. Post-optimization adjustments are often necessary to accommodate cultural preferences, market constraints, or regulatory requirements.

### 4.2 GAs in formulation

GAs are optimization methods inspired by natural selection and biological evolution (Holland, 1992). They have shown strong potential in exploring the complex formulation space involved in the development of functional foods. Rather than relying on exhaustive enumeration, GAs encode ingredient combinations and processing parameters as chromosomes. These chromosomes evolve through repeated cycles of selection, crossover, and mutation.

The search process is guided by a fitness function designed to meet predefined objectives, such as maximizing the maximum fiber content or reducing saturated fat levels (Liang et al., 2022; Thakur et al., 2023). Through iterative refinement (a process of gradually improving solutions over multiple cycles), GAs improve candidate formulations until acceptable results are reached. Compared to traditional trial-and-error methods, this approach provides a more efficient and structured way to handle multivariable optimization tasks.

A practical application is provided by Liang et al. (2022), who used a genetic algorithm to optimize blended frying oil formulations (Table 1). The model aimed to reduce the cost, improve the composition of fatty acids, and extend the frying life. The optimized blends showed stability similar to that of palm oil, with a lower saturated fat content.

Despite their advantages, GAs are sensitive to how the fitness function is defined. Poorly specified objectives can result in suboptimal formulations. Additionally, GAs may not always find the global optimum and can require substantial computational resources when applied to large or complex datasets.

### 4.3 FL applications

FL provides a transparent and adaptable framework for optimizing functional food formulations, especially in contexts involving imprecise or sensory-dependent variables. Unlike binary systems that classify inputs as strictly true or false, FL accommodates degrees of truth, allowing the modeling of qualitative descriptors, such as ‘moderate sweetness’ or “slightly bitter” (Klir & Yuan, 1995; Zadeh, 1965). This makes it particularly effective during early stage product development, where sensory judgment often complements quantitative data (Vivek et al., 2019).

The process begins with fuzzification, converting numerical values, such as pH, fiber content, or bitterness intensity, into linguistic variables using membership functions (Kahraman, 2008). These inputs are then processed through rule-based systems (*e.g.*, “If sweetness is high and fiber is low, then acceptability is moderate”). Finally, defuzzification translates the results into precise outputs to guide the formulation. This sequence supports decision-making even when the data are vague, nonlinear, or contradictory, offering a practical alternative to rigid parametric models (Birle et al., 2013).

The applications of FL span various domains. In a study conducted by Güldane (2023), a Mamdani-type fuzzy inference system, combined with experimental design of Taguchi, was used to optimize foam stability in a soy protein-based formulation. As a result, stability increased from 62.5 to 120 min after applying the FL model (Güldane, 2023). In another case, a Takagi-Sugeno fuzzy algorithm improved the sensory attributes of a mixed fruit beverage by integrating panel feedback with complex ingredient data (Dhar et al., 2011). These examples demonstrate how FL can take into account both sensory and technical factors in formulation decisions.

However, conventional FL systems depend on expert-defined rules and static membership functions, which may introduce bias and limit scalability (Y. Zhang et al., 2024). In contrast to data-driven models, they do not automatically learn from new information unless embedded in adaptive frameworks, such as neuro-fuzzy systems (Voloşencu, 2024). However, in product development scenarios that require transparency, expert oversight, or regulatory compliance, FL remains a valuable tool. Its ability to integrate nutritional, sensory, and technological considerations reinforces its relevance in the optimization of functional foods.

### 4.4 Heuristic search techniques

Heuristic search techniques have gained attention in functional food formulation. This is attributed to their ability to efficiently solve complex, nonlinear, and multi-objective problems as they mimic certain elements of human reasoning (Alvarez, 2020; Gigerenzer & Gaissmaier, 2011). Unlike exhaustive algorithms that test every solution, heuristics apply informed rules to reach acceptable results under time and resource constraints (Vuppalapati, 2022). This makes them suitable for balancing nutritional content, sensory properties, and production feasibility. Heuristic approaches are based on different conceptual models, evolution, thermodynamics, and collective intelligence; they are all capable of navigating large formulation spaces without full prior knowledge (Dorigo & Socha, 2018; Mitchell, 1998; D. Wang et al., 2017). This adaptability is particularly useful in food systems with undefined or nonlinear ingredient interactions.

Several studies highlight the practical value of these methods. Ming et al. (2021) applied a Particle Swarm Optimization (PSO)-enhanced artificial neural network (ANN) to optimize the spray drying of coconut milk. The improved model improved the prediction of quality traits while reducing the number of experimental trials. Similarly, Dubey and Tripathy (2024) used an ANN-PSO model to refine the hydration conditions for finger millet. This method reduced antinutrient levels and improved functional properties, such as emulsification and water absorption. It also induced structural changes in proteins, specifically increased β-sheet and random coils, suggesting a link between molecular transitions and functional outcomes.

However, heuristic models are nondeterministic and sensitive to parameter tuning (Mahmud et al., 2022). As a result, they may converge to locally optimal solutions (which are satisfactory only within a limited range) without reaching the globally optimal solution (which represents the best possible outcome across the entire solution space) (Karimi-Mamaghan et al., 2022). Therefore, this limitation can be critical in food-related applications that require precision, such as nutrient optimization or formulation standardization.

### 4.5 Classical ML models

Classical ML models, including RF, SVM, ANN, and Partial Least Squares Regression (PLSR), have been applied in optimization and formulation tasks in functional foods (Caporaso et al., 2018). These approaches are often used with moderately sized datasets to identify relationships between processing variables and functional outcomes. Their ability to integrate chemical, spectroscopic, and sensory data makes them useful tools for guiding process adjustments without the computational demands of DL. For example, Caporaso et al. (2018) employed PLSR and principal component regression to predict the fermentation index, total polyphenols, and antioxidant activity of cocoa beans using hyperspectral imaging (1000–2500 nm). The PLSR models achieved external validation coefficients (R^2^) of 0.50, 0.70, and 0.74 for fermentation index, total polyphenols, and antioxidant activity, respectively, sufficient for screening and process-monitoring purposes. Moreover, the models enabled chemical imaging of bioactive compound distribution within and between single beans. These findings illustrate how classical regression-based ML methods can integrate spectroscopic and biochemical data to support non-destructive functional assessment and guide quality optimization in food processing.

### 4.6 DL approaches

DL approaches are increasingly used for optimization and formulation, especially when input-output relationships are highly non-linear and exceed the capabilities of classical ML (Ma et al., 2023). These methods are especially valuable when large spectroscopic or omics datasets are available as they enable the capture of subtle interactions among compositional and processing factors influencing functional properties.

To illustrate these capabilities**,** Zhang, Jiang, et al. (2025) used a deep neural network (DNN) to optimize anthocyanin extraction from mulberries using an enzyme-ultrasound-assisted process. A Box–Behnken design was applied to assess the effects of solid-liquid ratio, ethanol concentration, ultrasonic temperature, and pectinase dosage. The DNN achieved higher predictive accuracy, with an R^2^ of 0.990 and a relative error of 0.85%, compared with the response surface methodology (RSM), which showed 0.940 and 4.50%, respectively. This indicates better fitting of nonlinear relationships among processing variables and extraction outcomes. The optimized conditions (50 ml.g^−1^ solid-liquid ratio, 63% ethanol, 40 °C ultrasonic temperature, and 0.5% pectinase) resulted in 3.16 mg.g-1 total anthocyanins and notable antioxidant activity (ABTS 98%, DPPH 80%, •OH 54%).

### 4.7 Hybrid and integrative strategies

Integrative strategies in optimization and formulation combine different computational techniques or data sources to enhance predictive reliability and identify optimal processing conditions**.** These approaches are particularly useful when a single model cannot capture the complexity of food systems (Dubey & Tripathy, 2024). This complexity often arises from non-linear interactions among variables or from the need to balance multiple outcomes, such as bioactive retention and sensory quality (Squara et al., 2024). In this regard, Dubey and Tripathy (2024) applied a hybrid ANN-PSO approach to optimize ultrasound-assisted extraction of bioactive compounds from finger millet. The ANN-PSO model achieved lower prediction errors (<10%) compared with RSM and identified optimum conditions (76% ultrasound amplitude, W:G 3.5:1, 17.5 min, 40 kHz) that enhanced phenolic content and antioxidant activity.

In a related effort, Younis et al. (2019) showed how hybrid AI strategies can integrate classical ML algorithms with optimization frameworks to enhance functional food processing. The authors used ANN, Gaussian process regression (GPR), and SVM models to model and optimize the de-bittering process of mosambi (*Citrus limetta*) peel powder. The ANN and GPR models achieved high predictive accuracy for total polyphenols (TPs) and antioxidant activity (AA) (R^2^ > 0.99), while the SVM classifier captured sensory acceptability patterns. Integration with a GA facilitated multi-objective optimization, identifying approximately 10% NaCl concentration and 7.9 h soaking time as optimal conditions balancing bioactive retention (TP ∼0.77%; AA ∼60%) with acceptable sensory quality.

## 5 AI-driven strategies for enhancing stability and bioavailability

Physicochemical instability during processing and gastrointestinal stresses, such as low pH and enzymatic degradation, adversely affect the stability and structure of bioactive compounds. This may lead to reduced bioavailability and, hence, reduced functional effectiveness. Therefore, ML techniques provide promising tools to address these challenges by optimizing delivery systems, simulating physiological conditions, and predicting molecular interactions (Aliakbarian et al., 2018; Espinosa Sandoval et al., 2023; Kundu et al., 2015; Yousefi & Razavi, 2017).

In encapsulation, ANNs have been applied to refine microencapsulation parameters, such as microencapsulation yield, moisture content, TP content, and encapsulation efficiency, for sensitive compounds. In their study, Aliakbarian et al. (2018) combined ANNs with response surface methodology to improve the spray drying of olive pomace polyphenols in order to enhance their stability. However, Kundu et al. (2015) developed a hybrid model integrating a neural network with a GA, supported by response surface methodology. In this system, the neural network was used to generate the initial population for the GA, while the RSM-derived equation served as the fitness function. This hybrid model optimized key parameters, including oil and surfactant concentrations, stirring speed, and stirring time, resulting in enhanced emulsion stability, as measured by the emulsion stability index. These modeling strategies support the design of delivery systems that protect labile compounds and regulate their release profiles. Therefore, future models may benefit from combining real-time sensor inputs, which would allow dynamic control during the encapsulation of heat-sensitive bioactive components. Furthermore, AI models have been used to simulate gastrointestinal environments to estimate the release and absorption of encapsulated nutrients. These models account for digestion time, gel volume, and concentration, with gel concentration emerging as the most influential factor. For example, Yousefi and Razavi (2017) predicted glucose release from starch gels using the adaptive neuro-fuzzy inference system, a GA-ANN model, and the group method of data handling.

In the future, AI can be combined with smart delivery technologies, such as stimuli-responsive carriers. This could advance the formulation of functional foods with improved bioavailability. However, these advances require high-quality data, validated models, and transparency to ensure regulatory compliance. Experimental validation remains essential to confirm predictive outcomes, and support safe and effective implementation.

## 6 AI in market trends and consumer behavior

Revealing behavioral patterns and aligning with regulatory frameworks are essential for anticipating demand and guiding functional food innovation. This section attempts to explore how AI facilitates consumer trend analysis and supports the substantiation of health claims, ensuring both market relevance and scientific credibility.

### 6.1 Predicting market trends and consumer preferences

Unlike traditional market research, AI can process large-scale, unstructured datasets (such as social media interactions, consumer reviews, and purchase logs) to extract meaningful insights. In this sense, ML algorithms are particularly effective in detecting complex, and nonlinear associations between product features and consumer values (X. Wang & Hu, 2024).

**Fig. 4:**
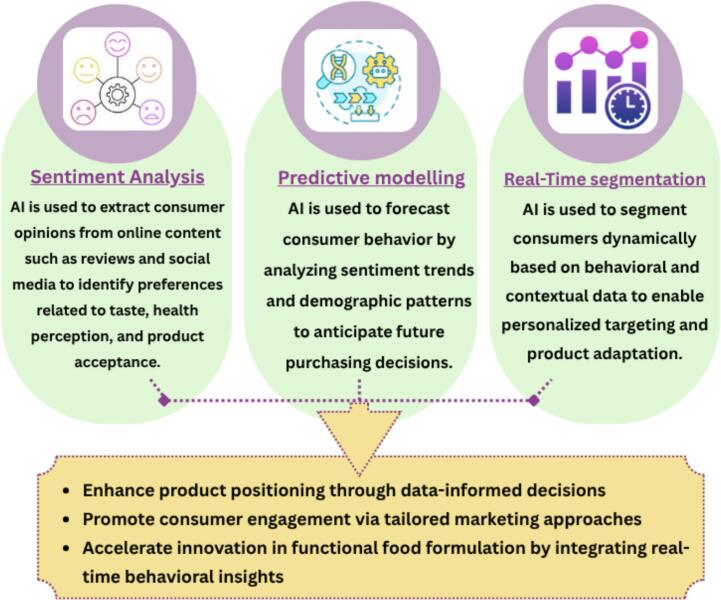
Use of AI-powered tools, such as sentiment analysis, predictive modeling, and real-time segmentation, to enable an understanding of consumer behavior. AI: artificial intelligence.

These capabilities allow manufacturers to develop products that are tailored to specific health interests and sustainability preferences. For example, AI enables real-time segmentation of consumers based on behavioral trends and wellness goals (Vetrivel et al., 2024). Additionally, Gooljar et al. (2024) reported in their systematic review that combining sentiment analysis with predictive modeling also helps anticipate shifts in consumer attitudes, supporting proactive marketing and product positioning. To the best of our knowledge, no published research to date explicitly integrates sentiment analysis and predictive modeling within the context of functional foods and consumer behavior. We believe future work could explore how combining sentiment analysis, predictive modeling, and real-time segmentation may help functional food companies respond more effectively to evolving consumer preferences. Fig. 4 describes how AI can be used to anticipate consumer trends in functional foods. It highlights three key AI tools: sentiment analysis, predictive modeling, and real-time segmentation. These tools help companies understand shifting preferences, personalize product positioning, and enhance consumer engagement (Pahwa & Gupta, 2026; Vetrivel et al., 2024).

Additionally, unsupervised learning methods, such as clustering, have proven effective in identifying latent consumer segments. Unlike conventional statistical tools, K-means do not rely on strict distributional assumptions (they can work even when the data do not follow a normal pattern) which is often the case in real-world consumer behavior (Raykov et al., 2016). An excellent example of this is a study conducted in Sicily by Sgroi et al. (2024), which used K-means clustering to explore how consumer preferences and behaviors toward functional foods vary across local populations. The study highlights that factors, such as gender, age, income level, and the degree of urbanization, significantly influence both the likelihood and frequency of functional food consumption. The outcomes of this study may serve as a foundation for developing policy briefs that support regional health initiatives and guide targeted marketing strategies.

Furthermore, AI also contributes to improving operational efficiency. For example, Panda and Mohanty (2023) conducted a comparative study using various ML and DL regressors, including RF, Gradient Boosting, LightGBM, XGBoost, CatBoost, long short-term memory (LSTM), and Bi-LSTM, to forecast weekly food orders. Their results demonstrated the forecasting potential of DL models, with LSTM outperforming the other evaluated models across all error metrics. Further, the work reported the importance of incorporating temporal and contextual variables (*e.g.*, dates, holidays, and events) to enhance predictive accuracy. Although the outcomes of the model are promising, examining its performance under more complex real-world conditions, such as sudden changes in demand or limited data, is essential, to ensure that it is reliable and applicable in food service settings.

This review suggests that the value of AI in functional food markets remains limited, mainly because of: (1) Data quality: incomplete intake logs or misreported health status can skew demand forecasts and product design; (2) transparency matters, especially in DL models, which often function as black boxes, because if nutritionists and regulators cannot understand why an algorithm singles out a demographic, its recommendations lose credibility; and (3) ethical responsibility is equally critical: personalized offers must respect privacy and avoid exploiting health anxieties.

Moreover, the effectiveness of these models is significantly influenced by the social and cultural contexts in which they are implemented, where consumer choices are shaped by complex health and behavioral factors.

### 6.2 AI in health claim validation and regulation

The rapid evolution of the functional food market calls for regulatory tools that can verify health claims with precision, ensure compliance with health standards, and optimize time and resource use in claim validation and regulatory review processes. In the United States, the Nutrition Labelling and Education Act of 1990 authorizes the Food and Drug Administration (FDA) to approve health claims only when supported by significant scientific consensus (U.S. Food and Drug Administration, 2024). The agency also oversees nutrient content and structure/function claims, which refer to statements describing how a nutrient supports normal body structure or function. For example, “calcium helps maintain strong bones” illustrates a structure claim, whereas “fiber supports normal bowel function” illustrates a function claim, rather than linking the nutrient to disease risk reduction. In the European Union, Regulation (EC) No. 1924/2006 similarly requires that health-related claims be based on robust scientific evidence and presented transparently (European Commission, 2006). At the global level, Codex Alimentarius guidelines promote consistency, and precision in food labelling and labelling practices **(****Codex Alimentarius,** 2004**).**

Health claims generally describe a relationship between a food component and the reduced risk of a disease or health condition (U.S. Food and Drug Administration, 2024). Verifying these claims is crucial for public health and consumer trust. Although few studies examining the use of AI applications to validate health claims are available, the existence of research in other food-related fields, such as Rodrigues et al. (**2023**) in food advertising monitoring and Mezgec et al. (**2019**) in dietary assessment, that lays the groundwork for these applications suggest that AI may be explored in this area soon. Banovic Fuentes et al. (2024) analyzed 97 omega-3 fatty acid supplements and found that a majority of the claims aligned with the EU Health Claims Registry; however, several products displayed unauthorized or weakly supported statements. Although this example relates to supplements, it could also occur in functional food products.

Another approach presented by Bhatlawande et al. (2024), who developed a mobile-based AI application designed to extract ingredient information from packaged food using Optical Character Recognition *via* the Google Vision API. The extracted text is processed through a rule-based algorithm that matches keywords to a predefined dictionary of ingredient categories, such as allergens, lactose, onion, garlic, and nut-based contents. The algorithm identifies these components and flags them accordingly. Additionally, the system assesses specific nutritional attributes, such as sugar, carbohydrate, salt, and energy content, by computing their percentage against the recommended daily intake. The proposed categorization system achieved an accuracy of 84% and precision of 87%, demonstrating its reliability in identifying ingredient categories and assisting users in making informed dietary decisions (Bhatlawande et al., 2024). Likewise, food entities can use this approach to automatically assess the suitability of product labels and verify whether the listed ingredients or health-related claims align with relevant regulatory guidelines, such as those established by the FDA or equivalent authorities.

## 7 Challenges and ethical implications

We attempted to discuss the limitations of using AI in each section of the review. However, the present study still has major limitations that cannot be ignored regarding two main topics: (1) the difficulty of modeling complex biological systems, such as the human gut microbiota, and (2) the ethical challenges arising from personalized nutrition, particularly regarding data privacy and user autonomy.

### 7.1 Limitations of AI in modeling the human gut microbiota

Although AI techniques have substantially advanced, their application to dynamic biological systems, such as the human gut microbiota, remains constrained. This ecosystem is governed by complex interactions between microbial communities, host physiology, and metabolic pathways, which are further modulated by individual-specific factors, including genetics, diet, lifestyle, disease status, and medication use **(**Ezzamouri et al., 2021**;** Gomes et al., 2018**;** Marcos-Zambrano et al., 2021**)**. As a result, host-microbiota interactions are highly context-dependent and difficult to model using generalized computational frameworks.

Current AI models, particularly those trained on cross-sectional or static datasets, have limited capacity to capture the temporal and causal dynamics underlying host-microbiota interactions **(**Dakal et al., 2024**;** Marcos-Zambrano et al., 2021**)**. Although ML approaches are effective in identifying correlations within structured datasets, they are less suited to modeling long-term feedback processes, such as microbial adaptation or bidirectional host-microbe signaling (Pan et al., 2024). This limitation largely reflects the reliance on static representations that fail to track successive changes and reciprocal effects over time. Ng et al. (2023) emphasized the need for longer-term studies to adequately assess probiotic-induced microbiota changes in patients with major depressive disorders.

Efforts to address temporal complexity have begun. For example, Baranwal et al. (2022) applied LSTM networks to model microbial interactions and metabolite production in a synthetic gut community comprising 25 species. Their findings demonstrated improved predictive performance compared with that of traditional ecological models, such as the generalized Lotka–Volterra system. However, the authors noted that model interpretability remains a substantial challenge, even when supported by post-hoc explanation techniques, such as locally interpretable model-agnostic explanations and gradient-based methods (Baranwal et al., 2022). Moreover, the extent to which models trained on simplified microbial systems can be generalized to more complex, host-involving environments remains uncertain and requires further validation.

Complementary experimental evidence highlights the importance of dynamic monitoring frameworks. Wiese et al. (2024) used an advanced *in vitro* gut model incorporating a mucus agar layer and time-resolved sampling to examine luminal and mucosal microbiota responses alongside SCFA production. Their results showed that prebiotic compounds, including inulin and 2′-fucosyllactose, induced distinct time-dependent shifts in microbial composition and SCFA profiles, particularly within mucosal-associated communities. These findings emphasize the limitations of static modeling approaches and reinforce the need for temporally resolved data when applying AI to gut microbiota systems.

Another major limitation arises from considerable inter-individual variability in gut microbiota composition, which restricts the generalization of functional food effects. Formulations that enhance SCFA production in one individual may yield minimal or even adverse outcomes in another because of differences in microbial composition, enzymatic activity, and species-specific metabolic capacity (Cuciniello et al., 2023; H. Liu et al., 2022). Consequently, AI models trained on datasets with limited demographic or geographic diversity risk producing biased or non-generalizable predictions (Moreno-Indias et al., 2021). Furthermore, many current AI frameworks inadequately account for the ecological and evolutionary processes shaping microbial behavior. Mechanisms, such as microbial competition, dietary adaptation, cross-feeding, niche specialization, and horizontal gene transfer are rarely integrated into computational models, despite their central role in determining community resilience and functional outcomes (San Roman & Wagner, 2018; Z. Yang et al., 2024). As a result, model outputs may offer simplified representations that do not fully reflect microbial responses to environmental change.

While AI provides valuable tools for pattern recognition and hypothesis generation in microbiome research, it remains insufficient for producing predictive models grounded in biological mechanisms. Bridging this gap will require integrative strategies that combine AI with longitudinal data, multi-omics layers, and ecological theory. Such approaches are essential to capture the adaptive nature of host-microbiota interactions and support the development of functionally precise and personalized food interventions.

### 7.2 AI-driven personalized nutrition and ethics considerations

**Fig. 5:**
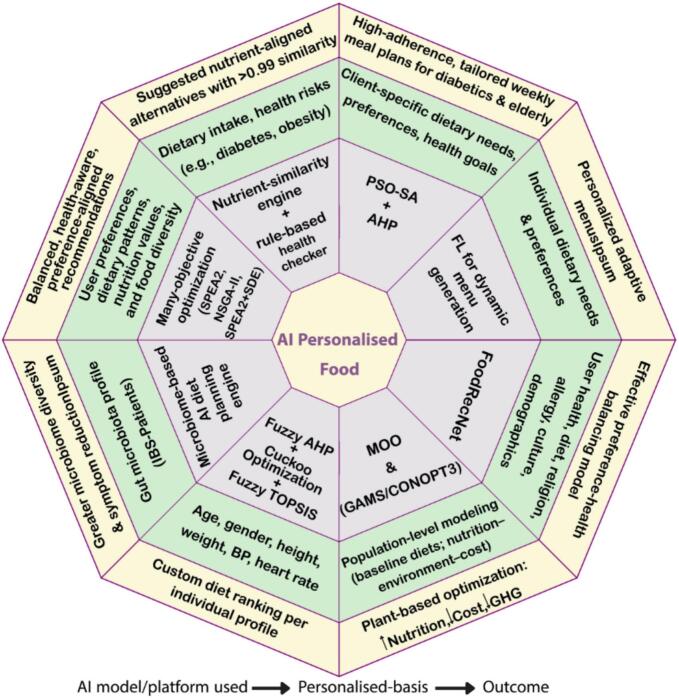
AI-driven approaches for functional food personalization, the inner layer: AI model or platform used, the middle layer: types of personalization basis, and the outer layer: personalized outcomes. AHP: analytic hierarchy process, AI: artificial intelligence; BP: blood pressure, CONOPT3: Constrained Optimization, version 3, FL: fuzzy logic, GAMS: General Algebraic Modeling System, IBS: irritable bowel syndrome, NSGA-II: Non-dominated Sorting Genetic Algorithm II, PSO-SA: Particle Swarm Optimization-Simulated Annealing, SDE: shift-based density estimation, SPEA: Strength Pareto Evolutionary Algorithm.

AI-driven personalized nutrition represents a move away from static dietary guidelines toward data-driven, individualized interventions. Fig. 5 displays eight research papers (Table A.2), each using different AI models/platforms, applied to various personalization criteria, along with their corresponding outcomes (outer layer). As summarized in Table A.2 and visualized in Fig. 5, recent studies apply diverse AI models and platforms to integrate microbiome data, clinical indicators, and behavioral variables for personalized dietary decision-making (Feng et al., 2023; Olutunde et al., 2024; Tunali et al., 2024; J. Zhang et al., 2022). These applications address a range of health contexts, including diabetes management, irritable bowel syndrome, and metabolically guided nutrition planning.

Fig. 5 indicates that most reported implementations rely on hybrid or multi-criteria decision-making frameworks, particularly when personalization involves clinical constraints and user preferences. This pattern is supported by hybrid systems combining PSO, simulated annealing, and Analytic Hierarchy Process for personalized meal planning in elderly individuals and patients with diabetes, which reported improved adherence in chronic disease contexts (Sarani Rad et al., 2024). However, Table A.2 also shows that empirical validation remains largely confined to controlled or high-resource settings. Several interventions are based on pilot studies, protocols, or short-term trials, including workplace-based evaluations of AI-driven dietary feedback applications designed to assess improvements in diet quality and cardiometabolic indicators. Long-term behavioral effects and clinical outcomes therefore remain insufficiently characterized across diverse populations (Ciecierski-Holmes et al., 2022; Kassem et al., 2025). Ethical and governance considerations further limit large-scale deployment. AI-driven personalized nutrition systems depend on continuous collection of sensitive personal data, including genetic, microbiome, metabolic, and behavioral information, which complicates anonymization and increases re-identification risks (Elendu et al., 2023; Olatunji et al., 2022; Rouskas et al., 2025; Sepas et al., 2022; Tsolakidis et al., 2024). These risks are amplified by decentralized data collection through mobile applications and wearable devices, often involving third-party platforms with limited transparency regarding data ownership and secondary use (Abrahams & Raimundo, 2025; Dias et al., 2022; Huhn et al., 2022; Pendergast et al., 2017; Puri et al., 2021).

Additional ethical challenges arise from cross-border data flows, where regulatory frameworks, such as the General Data Protection Regulation and the Health Insurance Portability and Accountability Act may not be consistently enforced (Bradford et al., 2020). Moreover, many AI-based nutrition systems operate as opaque models, limiting user understanding of how personal data inform recommendations and potentially undermining informed consent and trust (Detopoulou et al., 2023; Di Bitonto et al., 2024).

In light of these observations, the patterns summarized in Table A.2 and Fig. 5 suggest that future research should balance technical innovation with ethical robustness. Approaches, such as federated learning and XAI may help mitigate privacy and transparency concerns, but their effective adoption may depend on parallel advances in data governance, regulatory harmonization, and user-centered consent mechanisms.

## 8 Conclusion and critical perspective

This review attempts to map the current landscape of data-driven approaches in functional food research. AI accelerates bioactive discovery, improves metabolomic interpretation, and optimizes formulation design, and enhances the prediction of consumer behavior regarding functional products. The most consistent evidence emerges in predicting antioxidant activity and classifying bioactive compounds in fruit- and vegetable-based matrices, while cereals and marine resources remain notably underexplored. Considerable progress has been achieved by linking ML and DL models to curated bioactivity databases, such as ChEMBL, FooDB, and PubChem BioAssay, which enable data harmonization and compound prioritization. The integration of graph-based and NLP tools further bridges molecular data with health outcomes by extracting relationships from vast, unstructured knowledge sources. Despite these advances, several challenges remain, including dataset imbalance, inconsistent annotation, and the limited interpretability of black-box models, all of which hinder biological validation and regulatory translation.

Furthermore, unresolved ethical and privacy concerns related to personalized health data remain a major challenge as many studies are constrained by limited dataset size and diversity. Advancing this field requires developing standardized, multi-institutional datasets and promoting validation frameworks that bridge computational predictions with *in vitro*, *in vivo*, and clinical evidence. Meanwhile, XAI frameworks show growing potential by visualizing model reasoning and highlighting influential molecular or compositional features, thereby enhancing mechanistic interpretation, reproducibility, and transparency. AI-assisted functional food innovation must embed explainability and validation at all modeling stages while expanding matrix-specific datasets, particularly for cereals and marine products. Moreover, the adoption of multimodal AI approaches that integrate multi-omics data with spectroscopy and imaging may enhance the accuracy of predictions related to bioavailability, stability, and sensory attributes. Ethical and regulatory preparedness should also progress through privacy-preserving and equitable data governance to ensure reliable and responsible applications of AI in food science. Overall, the synergy between AI and food science has substantial potential, provided that future systems remain explainable, validated, and equitably designed to benefit diverse populations.

## CRediT authorship contribution statement

**Nadia Alkalbani:** Writing – original draft, Supervision, Project administration, Investigation, Formal analysis, Data curation, Conceptualization. **Leen Shahin:** Visualization, Data curation. **Hiba Benzeghiba:** Visualization, Data curation. **Reyad S. Obaid:** Writing – review & editing, Validation. **Tareq M. Osaili:** Writing – review & editing, Validation. **Leila Cheik Ismail:** Writing – review & editing. **Ghayah Al qasssimi:** Writing – review & editing. **Maha Rauf:** Writing – review & editing. **Khawla Abdulrahim:** Writing – review & editing. **Afra Almashgouni:** Writing – review & editing. **Fatima Ashuweihi:** Writing – review & editing. **Dana AL-Fuqaha:** Writing – review & editing.

## Declaration of competing interest

The authors declare that they have no known competing financial interests or personal relationships that could have appeared to influence the work reported in this paper.

## Data Availability

Data will be made available on request.

## Appendix A Supplementary data

Supplementary material Image 1

## References

[bb0005] Abejon R., Batlle-Bayer L., Laso J., Bala A., Vazquez-Rowe I., Larrea-Gallegos G., Aldaco R. (2020). Multi-objective optimization of nutritional, environmental and economic aspects of diets applied to the Spanish context. Foods.

[bb0010] Abrahams M., Raimundo M. (2025). Perspective on the ethics of AI at the intersection of nutrition and behaviour change. Frontiers in Aging.

[bb0015] Ahmad S., Al-Shabib N.A. (2020).

[bb0020] Aliakbarian B., Sampaio F.C., de Faria J.T., Pitangui C.G., Lovaglio F., Casazza A.A., Perego P. (2018). Optimization of spray drying microencapsulation of olive pomace polyphenols using response surface methodology and artificial neural network. LWT.

[bb0025] Alov P., Tsakovska I., Pajeva I. (2015). Computational studies of free radical-scavenging properties of phenolic compounds. Current Topics in Medicinal Chemistry.

[bb0030] Alvarez P.A., Kahraman C., Cebi S. (2020). Customer oriented product design: Intelligent and fuzzy techniques.

[bb0035] Amara A., Frainay C., Jourdan F., Naake T., Neumann S., Novoa-Del-Toro E.M., Witting M. (2022). Networks and graphs discovery in metabolomics data analysis and interpretation. Frontiers in Molecular Biosciences.

[bb0040] Astley S., Finglas P. (2016).

[bb0045] Ayyash M., Tarique M., Alaryani M., Al-Sbiei A., Masad R., Al-Saafeen B., Kamal-Eldin A. (2022). Bioactive properties and untargeted metabolomics analysis of bioaccessible fractions of non-fermented and fermented date fruit pomace by novel yeast isolates. Food Chemistry.

[bb0050] Banwo K., Olojede A.O., Adesulu-Dahunsi A.T., Verma D.K., Thakur M., Tripathy S., Utama G.L. (2021). Functional importance of bioactive compounds of foods with potential health benefits: A review on recent trends. Food Bioscience.

[bb0055] Baranwal M., Clark R.L., Thompson J., Sun Z., Hero A.O., Venturelli O.S. (2022). Recurrent neural networks enable design of multifunctional synthetic human gut microbiome dynamics. eLife.

[bb0060] Bashiri B., Kaleda A., Vilu R. (2025). Integrating multi-criteria decision-making with multi-objective optimization for sustainable diet design. Journal of Cleaner Production.

[bb0065] Bennett-Lenane H., Griffin B.T., O’Shea J.P. (2022). Machine learning methods for prediction of food effects on bioavailability: A comparison of support vector machines and artificial neural networks. European Journal of Pharmaceutical Sciences.

[bb0070] Bhatlawande S., Shilaskar S., Surana A. (2024). A smart scanner system for ingredient categorization and identification of nutritional composition in packaged food items. Journal of Integrated Science and Technology.

[bb0075] Birle S., Hussein M.A., Becker T. (2013). Fuzzy logic control and soft sensing applications in food and beverage processes. Food Control.

[bb0080] Bradford L., Aboy M., Liddell K. (2020). International transfers of health data between the EU and USA: A sector-specific approach for the USA to ensure an “adequate” level of protection. Journal of Law and the Biosciences.

[bb0085] Breda L.S., de Melo Nascimento J.E., Alves V., Arnaut d.A., de Toledo V., de Lima V.A., Felsner M.L. (2024). Green and fast prediction of crude protein contents in bee pollen based on digital images combined with random Forest algorithm. Food Research International.

[bb0090] Canatan M., Alkhulaifi N., Watson N., Boz Z. (2025). Artificial intelligence in Food manufacturing: A review of current work and future opportunities. Food Engineering Reviews.

[bb0095] Capanoglu E., Feng S., Janaswamy S. (2024). Editorial: Functional foods: Adding value to food. Frontiers in Nutrition.

[bb0100] Caporaso N., Whitworth M.B., Fowler M.S., Fisk I.D. (2018). Hyperspectral imaging for non-destructive prediction of fermentation index, polyphenol content and antioxidant activity in single cocoa beans. Food Chemistry.

[bb0105] Chang J., Wang H., Su W., He X., Tan M. (2025). Artificial intelligence in food bioactive peptides screening: Recent advances and future prospects. Trends in Food Science & Technology.

[bb0110] Chhetri K.B. (2023). Applications of artificial intelligence and machine learning in Food quality control and safety assessment. Food Engineering Reviews.

[bb0115] Chi J., Shu J., Li M., Mudappathi R., Jin Y., Lewis F., Gu H. (2024). Artificial intelligence in metabolomics: A current review. Trends in Analytical Chemistry.

[bb0120] Ciecierski-Holmes T., Singh R., Axt M., Brenner S., Barteit S. (2022). Artificial intelligence for strengthening healthcare systems in low- and middle-income countries: A systematic scoping review. npj Digital Medicine.

[bb0125] Codex Alimentarius C., Codex Alimentarius commission (2004). Guidelines for use of nutrition and health claims.

[bb0130] Cuciniello R., Di Meo F., Filosa S., Crispi S., Bergamo P. (2023). The antioxidant effect of dietary bioactives arises from the interplay between the physiology of the host and the gut microbiota: Involvement of short-chain fatty acids. Antioxidants.

[bb0135] Dakal T.C., Xu C., Kumar A. (2024). Advanced computational tools, artificial intelligence and machine-learning approaches in gut microbiota and biomarker identification. Frontiers in Medical Technology.

[bb0140] Delgado A., Issaoui M., Vieira M.C., Saraiva de Carvalho I., Fardet A. (2021). Food composition databases: Does it matter to human health?. Nutrients.

[bb0145] Detopoulou P., Voulgaridou G., Moschos P., Levidi D., Anastasiou T., Dedes V., Papadopoulou S.K. (2023). Artificial intelligence, nutrition, and ethical issues: A mini-review. Clinical Nutrition Open Science.

[bb0150] Dhar R., Bhalerao P.P., Chakraborty S. (2021). Formulation of a mixed fruit beverage using fuzzy logic optimization of sensory data and designing its batch thermal pasteurization process. Journal of Food Science.

[bb0155] Dhar R., Sagesser R., Weikert C., Yuan J., Wagner A. (2011). Adaptation of Saccharomyces cerevisiae to saline stress through laboratory evolution. Journal of Evolutionary Biology.

[bb0160] Di Bitonto P., Magarelli M., Novielli P., Romano D., Diacono D., de Trizio L., Tangaro S. (2024). From data to nutrition: The impact of computing infrastructure and artificial intelligence. Exploration of Foods and Foodomics.

[bb0165] Dias S.B., Oikonomidis Y., Diniz J.A., Baptista F., Carnide F., Bensenousi A., Hadjileontiadis L.J. (2022). Users’ perspective on the AI-based smartphone PROTEIN app for personalized nutrition and healthy living: A modified technology acceptance model (mTAM) approach. Frontiers in Nutrition.

[bb0170] Dickinson E., Rusilowicz M.J., Dickinson M., Charlton A.J., Bechtold U., Mullineaux P.M., Wilson J. (2018). Integrating transcriptomic techniques and k-means clustering in metabolomics to identify markers of abiotic and biotic stress in Medicago truncatula. Metabolomics.

[bb0175] Doherty A., Wall A., Khaldi N., Kussmann M. (2021). Artificial intelligence in functional Food ingredient discovery and characterisation: A focus on bioactive plant and Food peptides. Frontiers in Genetics.

[bb0180] Dong F., Hao J., Luo R., Zhang Z., Wang S., Wu K., Liu M. (2022). Identification of the proximate geographical origin of wolfberries by two-dimensional correlation spectroscopy combined with deep learning. Computers and Electronics in Agriculture.

[bb0185] Dorigo M., Socha K., Gonzalez T.F. (2018). Handbook of approximation algorithms and metaheuristics.

[bb0190] Dubey A., Tripathy P.P. (2024). Ultrasound-mediated hydration of finger millet: Effects on antinutrients, techno-functional and bioactive properties, with evaluation of ANN-PSO and RSM optimization methods. Food Chemistry.

[bb0195] Ekrami M., Shokrollahi Yancheshmeh B., Roshani-Dehlaghi N., Mobahi N., Emam-Djomeh Z., Mohammadifar M. (2025). Next-generation smart and safe foods: Artificial intelligence -driven strategies for 4D food pre-printing challenges. Trends in Food Science & Technology.

[bb0200] Elendu C., Amaechi D.C., Elendu T.C., Jingwa K.A., Okoye O.K., John Okah M., Alimi H.A. (2023). Ethical implications of AI and robotics in healthcare: A review. Medicine (Baltimore).

[bb0205] Espinosa Sandoval L.A., Polanía Rivera A.M., Castañeda Florez L., García Figueroa A., Cerqueira M.Â.P.R., Pastrana Castro L.M. (2023). Food structure engineering and Design for Improved Nutrition, health and well-being.

[bb0210] European Commission (2006).

[bb0215] Ezzamouri B., Shoaie S., Ledesma-Amaro R. (2021). Synergies of systems biology and synthetic biology in human microbiome studies. Frontiers in Microbiology.

[bb0220] Feng J., Liu H., Mai S., Su J., Sun J., Zhou J., Zhang Y., Wang Y., Wu F., Zheng G., Zhu Z. (2023). Protocol of a parallel, randomized controlled trial on the effects of a novel personalized nutrition approach by artificial intelligence in real world scenario. BMC Public Health.

[bb0225] Ferraz de Arruda H., Aleta A., Moreno Y. (2023). Food composition databases in the era of big data: Vegetable oils as a case study. Frontiers in Nutrition.

[bb0230] Flores Martinez K.E., Bloszies C.S., Bolino M.J., Henrick B.M., Frese S.A. (2024). Hemp hull fiber and two constituent compounds, N-trans-caffeoyltyramine and N-trans-feruloyltyramine, shape the human gut microbiome in vitro. Food Chemistry: X.

[bb0235] Ganje M., Jafari S.M., Tamadon A.M., Niakosari M., Maghsoudlou Y. (2019). Mathematical and fuzzy modeling of limonene release from amylose nanostructures and evaluation of its release kinetics. Food Hydrocolloids.

[bb0240] Gao S., Chau H.Y.K., Wang K., Ao H., Varghese R.S., Ressom H.W. (2022). Convolutional neural network-based compound fingerprint prediction for metabolite annotation. Metabolites.

[bb0245] Garg N., Sethupathy A., Tuwani R., Dokania S., Iyer A., Gupta A., Bagler G. (2017). FlavorDB: A database of flavor molecules. Nucleic Acids Research.

[bb0250] Gigerenzer G., Gaissmaier W. (2011). Heuristic decision making. Annual Review of Psychology.

[bb0255] Gomes A.C., Hoffmann C., Mota J.F. (2018). The human gut microbiota: Metabolism and perspective in obesity. Gut Microbes.

[bb0260] Gonzalez G., Gong S., Laponogov I., Bronstein M., Veselkov K. (2021). Predicting anticancer hyperfoods with graph convolutional networks. Human Genomics.

[bb0265] Gooljar V., Issa T., Hardin-Ramanan S., Abu-Salih B. (2024). Sentiment-based predictive models for online purchases in the era of marketing 5.0: A systematic review. Journal of Big Data.

[bb0270] Gorbachev V., Nikitina M., Velina D., Mutallibzoda S., Nosov V., Korneva G., Nikitin I. (2022). Artificial neural networks for predicting Food antiradical potential. Applied Sciences.

[bb0275] Gu C., Wang G., Zhuang W., Hu J., He X., Zhang L., Du Z., Xu X., Yin M., Yao Y., Sun X., Hu W. (2026). Artificial intelligence-enabled analysis methods and their applications in food chemistry. Critical Reviews in Food Science and Nutrition.

[bb0280] Gu M., Li C., Chen L., Li S., Xiao N., Zhang D., Zheng X. (2023). Insight from untargeted metabolomics: Revealing the potential marker compounds changes in refrigerated pork based on random forests machine learning algorithm. Food Chemistry.

[bb0285] Guardado Yordi E., Koelig R., Matos M.J., Pérez Martínez A., Caballero Y., Santana L., Uriarte E. (2019). Artificial intelligence applied to flavonoid data in Food matrices. Foods.

[bb0290] Güldane M. (2023). Optimizing foam quality characteristics of model food using Taguchi-based fuzzy logic method. Journal of Food Process Engineering.

[bb0295] Gygi J.P., Kleinstein S.H., Guan L. (2023). Predictive overfitting in immunological applications: Pitfalls and solutions. Human Vaccines & Immunotherapeutics.

[bb0300] Holland J.H. (1992).

[bb0305] Hong M.J., Ko C.S., Kim J.B., Kim D.Y. (2025). Enhancement of the seed color, antioxidant properties, and agronomic traits of colored wheat via gamma radiation mutagenesis. Foods.

[bb0310] Hoseini B., Jaafari M.R., Golabpour A., Momtazi-Borojeni A.A., Eslami S. (2023). Optimizing nanoliposomal formulations: Assessing factors affecting entrapment efficiency of curcumin-loaded liposomes using machine learning. International Journal of Pharmaceutics.

[bb0315] Hu H., Mei Y., Wei Y., Xu Z., Zhao Y., Xu H., Mao X., Huang L. (2024). Chemical composition prediction in goji (Lycium barbarum) using hyperspectral imaging and multi-task 1DCNN with attention mechanism. LWT.

[bb0320] Huhn S., Axt M., Gunga H.C., Maggioni M.A., Munga S., Obor D., Barteit S. (2022). The impact of wearable Technologies in Health Research: Scoping review. JMIR mHealth and uHealth.

[bb0325] Jiang S., Mo F., Li W., Yang S., Li C., Jiang L. (2025). Deep learning-driven optimization of antihypertensive properties from whey protein hydrolysates: A multienzyme approach. Journal of Agricultural and Food Chemistry.

[bb0330] Jones P.J., Jew S. (2007). Functional food development: Concept to reality. Trends in Food Science & Technology.

[bb0335] Kahraman C. (2008).

[bb0340] Karimi-Mamaghan M., Mohammadi M., Meyer P., Karimi-Mamaghan A.M., Talbi E.-G. (2022). Machine learning at the service of meta-heuristics for solving combinatorial optimization problems: A state-of-the-art. European Journal of Operational Research.

[bb0345] Kassem H., Beevi A.A., Basheer S., Lutfi G., Cheikh Ismail L., Papandreou D. (2025). Investigation and assessment of AI’S role in nutrition-an updated narrative review of the evidence. Nutrients.

[bb0350] Khan R.S., Grigor J., Winger R., Win A. (2013). Functional food product development – Opportunities and challenges for food manufacturers. Trends in Food Science & Technology.

[bb0355] Klir G.J., Yuan B. (1995).

[bb0360] Koraqi H., Wawrzyniak J., Aydar A.Y., Pandiselvam R., Khalide W., Petkoska A.T., Rustagi S. (2025). Application of multivariate analysis and Kohonen neural network to discriminate bioactive components and chemical composition of kosovan honey. Food Control.

[bb0365] Kundu P., Paul V., Kumar V., Mishra I.M. (2015). Formulation development, modeling and optimization of emulsification process using evolving RSM coupled hybrid ANN-GA framework. Chemical Engineering Research and Design.

[bb0370] Lacroix S., Klicic Badoux J., Scott-Boyer M.-P., Parolo S., Matone A., Priami C., Moco S. (2018). A computationally driven analysis of the polyphenol-protein interactome. Scientific Reports.

[bb0375] Li W., Liu X., Liu Y., Zheng Z. (2025). High-accuracy identification and structure-activity analysis of antioxidant peptides via deep learning and quantum chemistry. Journal of Chemical Information and Modeling.

[bb0380] Li Y., Li X., Liu Y., Yao Y., Huang G. (2022). MPMABP: A CNN and bi-LSTM-based method for predicting multi-activities of bioactive peptides. Pharmaceuticals (Basel).

[bb0385] Liang J., Lim K., Niu F., Xia T., Jiang Y. (2022). Genetic algorithm predicts blended oil formulations with improved nutrition, prolonged frying life, and low cost. ACS Food Science & Technology.

[bb0390] Liu H., Liao C., Wu L., Tang J., Chen J., Lei C., Dai L. (2022). Ecological dynamics of the gut microbiome in response to dietary fiber. The ISME Journal.

[bb0395] Liu Y., Pu H., Li Q., Sun D.W. (2023). Discrimination of Pericarpium Citri Reticulatae in different years using terahertz time-domain spectroscopy combined with convolutional neural network. Spectrochimica Acta. Part A, Molecular and Biomolecular Spectroscopy.

[bb0400] Ma J., Zhou X., Xie B., Wang C., Chen J., Zhu Y., Huang F. (2023). Application for identifying the origin and predicting the physiologically active ingredient contents of Gastrodia elata Blume using visible–near-infrared spectroscopy combined with machine learning. Foods.

[bb0405] Magnini M., Ciatto G., Canturk F., Aydogan R., Omicini A. (2023). Symbolic knowledge extraction for explainable nutritional recommenders. Computer Methods and Programs in Biomedicine.

[bb0410] Mahmud S., Abbasi A., Chakrabortty R.K., Ryan M.J. (2022). A self-adaptive hyper-heuristic based multi-objective optimisation approach for integrated supply chain scheduling problems. Knowledge-Based Systems.

[bb0415] Malwe A.S., Srivastava G.N., Sharma V.K. (2023). GutBug: A tool for prediction of human gut Bacteria mediated biotransformation of biotic and xenobiotic molecules using machine learning. Journal of Molecular Biology.

[bb0420] Marcos-Zambrano L.J., Karaduzovic-Hadziabdic K., Loncar Turukalo T., Przymus P., Trajkovik V., Aasmets O., Truu J. (2021). Applications of machine learning in human microbiome studies: A review on feature selection, biomarker identification. Disease Prediction and Treatment. Front Microbiol.

[bb0425] Martorell-Marugan J., Tabik S., Benhammou Y., del Val C., Zwir I., Herrera F., Carmona-Saez P., Husi H. (2019). Computational biology.

[bb0430] Meima M.Y., Westerhout J., Bijlsma S., Meijerink M., Houben G.F. (2023). Coupling food compounds data from FooDB and phenol-explorer to the Dutch food coding system NEVO: Towards a novel approach to studying the role of food in health and disease. Journal of Food Composition and Analysis.

[bb0435] Mezgec S., Eftimov T., Bucher T., Korousic Seljak B. (2019). Mixed deep learning and natural language processing method for fake-food image recognition and standardization to help automated dietary assessment. Public Health Nutrition.

[bb0440] Ming J.L.K., Anuar M.S., How M.S., Noor S.B.M., Abdullah Z., Taip F.S. (2021). Development of an artificial neural network utilizing particle swarm optimization for modeling the spray drying of coconut Milk. Foods.

[bb0445] Mitchell M. (1998).

[bb0450] Mobahi N., Razavi M.A., Ekrami M., Emam-Djomeh Z., Razavi S.H. (2025). AI-integrated bio-nanocomposite food packaging: Sustainable, functional, and intelligent solutions. Trends in Food Science & Technology.

[bb0455] Moreno-Indias I., Lahti L., Nedyalkova M., Elbere I., Roshchupkin G., Adilovic M., Claesson M.J. (2021). Statistical and machine learning techniques in human microbiome studies: Contemporary challenges and solutions. Frontiers in Microbiology.

[bb0460] Ng Q.X., Lim Y.L., Yaow C.Y.L., Ng W.K., Thumboo J., Liew T.M. (2023). Effect of probiotic supplementation on gut microbiota in patients with major depressive disorders: A systematic review. Nutrients.

[bb0465] Olatunji I.E., Rauch J., Katzensteiner M., Khosla M. (2022). A review of anonymization for healthcare data. Big Data.

[bb0470] Olutunde T., Ani C.L., Adesue G.A. (2024). Leveraging machine learning for personalized dietary recommendations, nutritional patterns, and health outcome predictions. Journal of Science Research and Reviews.

[bb0475] Pahwa N., Gupta K., Madaan G., Singh A., Chahal B.P.S., David A., Singh G. (2026). Generative AI in Food systems: Predictive demand, smart supply chains, and sustainable service futures.

[bb0480] Pan R., Guo M., Chen Y., Lin G., Tian P., Wang L., Wang G. (2024). Dynamics of the gut microbiota and Faecal and serum metabolomes during pregnancy—A longitudinal study. Nutrients.

[bb0485] Panda S.K., Mohanty S.N. (2023). Time series forecasting and modeling of Food demand supply chain based on Regressors analysis. IEEE Access.

[bb0490] Park J., Beck B.R., Kim H.H., Lee S., Kang K. (2022). A brief review of machine learning-based bioactive compound research. Applied Sciences.

[bb0495] Pendergast F.J., Ridgers N.D., Worsley A., McNaughton S.A. (2017). Evaluation of a smartphone food diary application using objectively measured energy expenditure. International Journal of Behavioral Nutrition and Physical Activity.

[bb0500] Periwal V., Bassler S., Andrejev S., Gabrielli N., Patil K.R., Typas A., Patil K.R. (2022). Bioactivity assessment of natural compounds using machine learning models trained on target similarity between drugs. PLoS Computational Biology.

[bb0505] Puri V., Kataria A., Sharma V. (2021). Artificial intelligence-powered decentralized framework for internet of things in healthcare 4.0. Transactions on Emerging Telecommunications Technologies.

[bb0510] Qian J., Yu F., Arnold L.A., Saha A., Zheng L., Zhao M. (2024). Exploring structural features of sleep-enhancing peptides derived from casein hydrolysates by chemometrics and random forest methodology. Food Chemistry.

[bb0515] Rangarajan H., Hadka D., Reed P., Lynch J.P. (2022). Multi-objective optimization of root phenotypes for nutrient capture using evolutionary algorithms. The Plant Journal.

[bb0520] Ratanasanya S., Chindapan N., Polvichai J., Sirinaovakul B., Devahastin S. (2018). Particle swarm optimization as alternative tool to sensory evaluation to produce high-quality low-sodium fish sauce via electrodialysis. Journal of Food Engineering.

[bb0525] Raykov Y.P., Boukouvalas A., Baig F., Little M.A. (2016). What to do when K-means clustering fails: A simple yet principled alternative algorithm. PLoS One.

[bb0530] Rico D., Cano A.B., Alvarez Alvarez S., Rio Briones G., Martin Diana A.B. (2023). Study of the Total antioxidant capacity (TAC) in native cereal-pulse flours and the influence of the baking process on TAC using a combined Bayesian and support vector machine modeling approach. Foods.

[bb0535] Rodrigues M.B., Ferreira V.P., Claro R.M., Martins A.P.B., Avila S., Horta P.M. (2023). Revolutionising food advertising monitoring: A machine learning-based method for automated classification of food videos. Public Health Nutrition.

[bb0540] Rong L., Wang Y., Wang Y., Jiang D., Bai J., Wu Z., Li L., Wang T., Tan H. (2024). A fresh-cut papaya freshness prediction model based on partial least squares regression and support vector machine regression. Heliyon.

[bb0545] Rouskas K., Guela M., Pantoura M., Pagkalos I., Hassapidou M., Lalama E., Argiriou A. (2025). The influence of an AI-driven personalized nutrition program on the human gut microbiome and its health implications. Nutrients.

[bb0550] Sahin O., Aytekin-Sahin G. (2024). Open-source multi-objective optimization software for menu planning. Expert Systems with Applications.

[bb0555] Sakre N., Das A.B., Srivastav P.P. (2016). Fuzzy logic approach for process optimization of gluten-free pasta. Journal of Food Processing and Preservation.

[bb0560] Salahuddin Z., Woodruff H.C., Chatterjee A., Lambin P. (2022). Transparency of deep neural networks for medical image analysis: A review of interpretability methods. Computers in Biology and Medicine.

[bb0565] San Roman M., Wagner A. (2018). An enormous potential for niche construction through bacterial cross-feeding in a homogeneous environment. PLoS Computational Biology.

[bb0570] Sanchez-Reinoso Z., García-Vela S., Clément J.-P., Bazinet L. (2025). Combining statistical, machine learning and experimental approaches for screening of novel antimicrobial peptides of calf cruor hydrolysates. Food Bioscience.

[bb0575] Sarani Rad F., Amiri M., Li J. (2024). Optimizing nutritional decisions: A particle swarm optimization-simulated annealing-enhanced analytic hierarchy process approach for personalized meal planning. Nutrients.

[bb0580] Scalbert A., Brennan L., Manach C., Andres-Lacueva C., Dragsted L.O., Draper J., Wishart D.S. (2014). The food metabolome: A window over dietary exposure123. American Journal of Clinical Nutrition.

[bb0585] Sepas A., Bangash A.H., Alraoui O., El Emam K., El-Hussuna A. (2022). Algorithms to anonymize structured medical and healthcare data: A systematic review. Frontiers in Bioinformatics.

[bb0590] Sgroi F., Sciortino C., Baviera-Puig A., Modica F. (2024). Analyzing consumer trends in functional foods: A cluster analysis approach. Journal of Agriculture and Food Research.

[bb0595] Squara S., Caratti A., Fina A., Liberto E., Koljancic N., Spanik I., Cordero C. (2024). Artificial intelligence decision making tools in food metabolomics: Data fusion unravels synergies within the hazelnut (Corylus avellana L.) metabolome and improves quality prediction. Food Research International.

[bb0600] Sung J., Frost S., Suh J.H. (2025). Progress in flavor research in food: Flavor chemistry in food quality, safety, and sensory properties. Food Chemistry: X.

[bb0605] Thakur A., Panigrahi C., Mishra H.N. (2023). Formulation and characterization of a low-cost high-energy grain-based beverage premix: A multi-objective genetic algorithm approach. Food Chemistry Advances.

[bb0610] Topolska K., Florkiewicz A., Filipiak-Florkiewicz A. (2021). Functional Food—Consumer motivations and expectations. International Journal of Environmental Research and Public Health.

[bb0615] Tsolakidis D., Gymnopoulos L.P., Dimitropoulos K. (2024). Artificial intelligence and machine learning technologies for personalized nutrition: A review. Informatics.

[bb0620] Tunali V., Arslan N.C., Ermis B.H., Dervis Hakim G., Gundogdu A., Hora M., Nalbantoglu O.U. (2024). A multicenter randomized controlled trial of microbiome-based artificial intelligence-assisted personalized diet vs low-fermentable oligosaccharides, disaccharides, monosaccharides, and polyols diet: A novel approach for the management of irritable bowel syndrome. The American Journal of Gastroenterology.

[bb0630] Umaña M., Llull L., Bon J., Eim V.S., Simal S. (2022). Artificial neural networks to optimize oil-in-water emulsion stability with Orange by-products. Foods.

[bb0635] Uribe-Guerra G.D., Múnera-Ramírez D.A., Arias-Londoño J.D. (2024). Feed formulation using multi-objective Bayesian optimization. Computers and Electronics in Agriculture.

[bb0640] Vetrivel S.C., Saravanan T.P., Arun V.P., Maheswari R., Nadda V., Tyagi P.K., Singh A., Singh V. (2024). Integrating AI-driven technologies into service marketing.

[bb0645] Vivek K., Subbarao K.V., Routray W., Kamini N.R., Dash K.K. (2019). Application of fuzzy logic in sensory evaluation of Food products: A comprehensive study. Food and Bioprocess Technology.

[bb0650] Voloşencu C. (2024).

[bb0655] Vuppalapati C. (2022).

[bb0660] Wan T.T.H., Matthews S., Luh H., Zeng Y., Wang Z., Yang L. (2022). A proposed multi-criteria optimization approach to enhance clinical outcomes evaluation for diabetes care: A commentary. Health Services Research and Managerial Epidemiology.

[bb0665] Wang D., Tan D., Liu L. (2017). Particle swarm optimization algorithm: An overview. Soft Computing.

[bb0670] Wang H., Tian H., Ju R., Ma L., Yang L., Chen J., Liu F. (2024). Nutritional composition analysis in food images: An innovative Swin transformer approach. Frontiers in Nutrition.

[bb0675] Wang T., Holscher H.D., Maslov S., Hu F.B., Weiss S.T., Liu Y.Y. (2025). Predicting metabolite response to dietary intervention using deep learning. Nature Communications.

[bb0680] Wang X., Hu B. (2024). Machine learning algorithms for improved product design user experience. IEEE Access.

[bb0685] Wang Z., Huang Q., Ji S., Amrouche A.T., Zhu Y., Li X., Lu B. (2025). Personalized diets based on multi-objective optimization of nutrition and sensory characteristics: A digital strategy for enhancing food quality. Trends in Food Science & Technology.

[bb0690] Wang Z., Zhou L., Hao W., Liu Y., Xiao X., Shan X., Wei B. (2023). Comparative antioxidant activity and untargeted metabolomic analyses of cherry extracts of two Chinese cherry species based on UPLC-QTOF/MS and machine learning algorithms. Food Research International.

[bb0695] Wang Z.-K., Ta N., Wei H.-C., Wang J.-H., Zhao J., Li M. (2024). Research of 2D-COS with metabolomics modifications through deep learning for traceability of wine. Scientific Reports.

[bb0700] Westerman K.E., Harrington S., Ordovas J.M., Parnell L.D. (2020). PhyteByte: Identification of foods containing compounds with specific pharmacological properties. BMC Bioinformatics.

[bb0705] Wiese M., van der Wurff M., Ouwens A., van Leijden B., Verheij E.R., Heerikhuisen M., van der Vossen J.M.B.M. (2024). Modeling the effects of prebiotic interventions on luminal and mucosa-associated gut microbiota without and with Clostridium difficile challenge in vitro. Frontiers in Nutrition.

[bb0710] Yang G., Yu S., Sheng Y., Yang H. (2023). Attention and feature transfer based knowledge distillation. Scientific Reports.

[bb0715] Yang H., Jiao W., Zouyi L., Diao H., Xia S. (2025). Artificial intelligence in the food industry: Innovations and applications. Discover Artificial Intelligence.

[bb0720] Yang X., Zhu L., Jiang W., Yang Y., Niu L., Zhao Y., Wang Y., Chen L., Gan M., Zhu L., Shen L. (2025). Artificial intelligence-driven food quality prediction: Applying machine learning ensemble models for dynamic forecasting of pork pH and meat color changes. Food Chemistry.

[bb0725] Yang Z., Zhu J., Lu W., Tian F., Zhang H., Chen W. (2024). Integrating artificial intelligence in exploring multiscale gut microbiota and diet relations for health promotion: A comprehensive review. Food Bioscience.

[bb0730] Yikmis S., Turkol M., Pacal I., Duman Altan A., Tokatli N., Abdi G., Aadil R.M. (2025). Optimization of bioactive compounds and sensory quality in thermosonicated black carrot juice: A study using response surface methodology, gradient boosting, and fuzzy logic. Food Chemistry: X.

[bb0735] Younis K., Ahmad S., Osama K., Malik M.A. (2019). Optimization of de-bittering process of mosambi (*Citrus limetta*) peel: Artificial neural network, Gaussian process regression and support vector machine modeling approach. Journal of Food Process Engineering.

[bb0740] Yousefi A.R., Razavi S.M. (2017). Modeling of glucose release from native and modified wheat starch gels during in vitro gastrointestinal digestion using artificial intelligence methods. International Journal of Biological Macromolecules.

[bb0745] Zadeh L.A. (1965). Fuzzy sets. Information and Control.

[bb0750] Zhang C., Ding W., Mamattursun A., Ma X., Qi S., Wu Y., Zhang J., Ma X. (2025). Optimization of enzyme-ultrasound assisted extraction from mulberries anthocyanins based on response surface methodology and deep neural networks and analysis of in vitro antioxidant activities. Food Chemistry.

[bb0755] Zhang H., Jiang S., Sun H., Li Y., Yao Z. (2025). Exploration of novel antimicrobial agents against foodborne pathogens via a deep learning approach. Journal of Agricultural and Food Chemistry.

[bb0760] Zhang J., Li M., Liu W., Lauria S., Liu X. (2022). Many-objective optimization meets recommendation systems: A food recommendation scenario. Neurocomputing.

[bb0765] Zhang Y., Bao X., Zhu Y., Dai Z., Shen Q., Xue Y. (2024). Advances in machine learning screening of food bioactive compounds. Trends in Food Science & Technology.

[bb0770] Zhao Z., Kantono K., Kam R., Le T.T., Kitundu E., Chen T., Hamid N. (2025). Improving the bioactivities of apricot kernels through fermentation: Investigating the relationship between bioactivities, polyphenols, and amino acids through the Random Forest Regression XAI approach. Foods.

