# Supplementary material for: Artificial intelligence in functional food innovation: Bioactive enhancement and formulation optimization: A quasi-systematic review
Source: Food Chem X. 2026 Feb 7;34:103628. doi: 10.1016/j.fochx.2026.103628 (PMC12914456; doi:10.1016/j.fochx.2026.103628)
Supplement: Supplementary file 1 — Supplementary material [file mmc1.docx]

Article title: **Artificial Intelligence in Functional Food Innovation: Bioactive Enhancement and Formulation Optimization: A Quasi-Systematic Review**

Nadia Alkalbani ^1,2,*^, Leen Shahin^1^, Hiba Benzeghiba^1^, Reyad S. Obaid^1,2^, Tareq M. Osaili ^1,2,3^, Leila Cheik Ismail ^1,2,4^ Ghayah Al qasssimi ^1^, Maha Rauf ^1^, Khawla Abdulrahim ^1^, Afra Almashgouni ^1^, Fatima Ashuweihi ^1^, Dana AL-Fuqaha ^1^

1 Department of Clinical Nutrition and Dietetics, College of Health Sciences, University of Sharjah, Sharjah PO Box 27272, United Arab Emirates

2 Research Institute of Medical & Health Sciences, University of Sharjah, Sharjah PO. Box 27272, United Arab Emirates

3 Department of Nutrition and Food Technology, Faculty of Agriculture, Jordan University of Science and Technology, PO Box 3030, Irbid 22110

4 Nuffield Department of Women’s & Reproductive Health, University of Oxford, Oxford OX1 2JD, UK

* Correspondence: [nalkalbani@sharjah.ac.ae](mailto:nalkalbani@sharjah.ac.ae)

**Appendix A**

| **Table A.1**: Selected studies (2015-2025) on the application of artificial intelligence in the development of functional foods. | | | | | |
| --- | --- | --- | --- | --- | --- |
| **Food Matrix** | **AI Type** | **Model** | **Objective** | **Key Findings** | **Reference** |
| *Cynara cardunculus* (Cardoon) | Data-driven AI (classical ML) | RF, SVM, GB | Classify plant extracts and predict antimicrobial activity on FT-MIR spectra and compound profiles. | FT-MIR spectroscopy combined with ML enabled accurate prediction of antimicrobial potential, supporting drug discovery efforts. | (Sampaio & Calado, 2024) |
| None specified (503 food/herbal substances) | Data-driven AI (classical ML) | RF, SVM, GB | Identify COX-2 inhibitory compounds from food–medicine homologous substances using ML. | ML models predicted COX-2 and mPGES-1 inhibition, aiding anti-inflammatory compound screening. | (Tian *et al.*, 2023) |
| ***Anacardium occidentale* L. (**Cashew apple pomace) | Data-driven AI (classical ML) | GPR, SVR | Model the effect of drying temperature on bioactive compound content and antioxidant capacity of cashew pomace. | GPR-SVR models effectively predicted antioxidant levels; temperature had a major influence on compound retention. | (Luka *et al.*, 2022) |
| *Citrus limetta* (Mosambi peel powder) | Hybrid and integrative approaches | ANN, GPR, SVM | Optimize the debittering process to enhance polyphenol content, antioxidant activity, and sensory acceptability. | optimized process improved functional properties, suggesting potential for commercial application. | (Younis *et al.*, 2019) |
| *Sus scrofa domesticu* (Refrigerated pork muscle tissue) | Data-driven AI (classical ML) | RF | Identify marker compounds and metabolic pathway changes during cold storage using untargeted metabolomics. | AI identified 40 spoilage-related markers; pentose-related metabolism played a key role. | (Gu *et al.*, 2023) |
| Food-derived natural compounds | Hybrid and integrative approaches | BioDeepNat | Identify myocardial infarction-related protein targets of food-derived compounds in Asian populations. | Compounds such as resveratrol and kaempferol showed strong binding, influenced by genetic variations. | (Zhou *et al.*, 2024) |
| *Neptunia oleracea* Lour. (water mimosa) | Data-driven AI (classical ML) | RF, PLS, PLS-DA | Evaluate the relationship between phenolic content and antioxidant/antidiabetic activities. | Quercetin, kaempferol derivatives, and caffeic acid were key predictors of bioactivity; PLS outperformed RF. | (Lee *et al.*, 2018) |
| (Wine grape berries)  *Vitis vinifera* L. | Data-driven AI (classical ML) | GBM, RF | Predict skin flavonoid content using physical and mechanical berry traits. | GBM-RF ensemble model accurately predicted flavonoid levels, aiding grape selection. | (Brillante *et al.*, 2015) |
| *Haliotis diversicolor* (Abalone) | Data-driven AI (classical ML) | K-NN, RF | Predict tyrosinase-inhibitory peptides from abalone proteins using ML. | ML models identified peptides with strong inhibitory potential for natural enzyme inhibition. | (Kongsompong *et al.*, 2021) |
| *Gastrodia elata* blume | Deep learning | 1D-CNN | Predict geographical origin and active compound content using 1D-CNN and Vis-NIR spectroscopy. | Model accurately classified origin and compound levels, supporting quality control applications. | (Ma *et al.*, 2023) |
| Vigna unguiculata L. Walp. (Cowpea beans) | Data-driven AI (classical ML) | K-NN, PLS-DA, RF, SVM | Classify cowpea varieties based on elemental fingerprinting. | RF and SVM achieved high accuracy in varietal discrimination. | (Pérez-Rodríguez *et al.*, 2019) |
| *Psidium guajava* L. (Red-fleshed guava) | Data-driven AI (classical ML) | RF, GB | Characterize fruit quality traits and identify predictors of antioxidant activity. | Total phenols, β-carotene, and flavonoids strongly influenced antioxidant potential. | (Mishra *et al.*, 2022) |
| Momordica charantia (Bitter melon) | Deep learning | ANN | Predict bioactive compound accumulation under various elicitor treatments. | ANN effectively modelled compound levels, guiding bioactivity enhancement. | (Jandoust *et al.*, 2023) |
| cv. *Musa nana* and *Musa cavendishii* (Bananas) | Deep learning | ANN | Model effects of drying on antioxidant and phenolic content. | ANN accurately predicted the outcomes; lyophilization preserved more bioactives. | (Guiné *et al.*, 2015) |
| *Theobroma cacao* L. (Cocoa shells) | Deep learning | ANN | Optimize aqueous phenolic extraction from cocoa shells. | ANN and RSM accurately modelled extraction; acidity, temperature, and S/L ratio were key variables. | (Rebollo-Hernanz *et al.*, 2021) |
| Berberis vulgaris (Seedless barberry) | Knowledge-driven AI | Fuzzy logic | Compare extraction methods for optimizing bioactive recovery. | Subcritical water led to highest antioxidant activity; cold press and CO₂ also effective. | (Sharifi & Khoshnoudi-Nia, 2022) |
| *Nizimuddinia zanardini* (Iranian brown macroalgae) | Knowledge-driven AI | Fuzzy logic | Rank extraction methods for maximizing bioactive content. | Microwave-assisted extraction yielded the highest phenolics and antioxidant activity. | (Taherkhani *et al.*, 2024) |
| Fresh apple juice with added betel leaf (Piper betle L. ) essential oil (BLEO) | Knowledge-driven AI | Fuzzy logic | Determine optimal BLEO concentration for preservation and sensory quality. | BLEO at 0.19 µL/mL improved antioxidant levels and shelf-life by 6 days. | (Basak, 2018) |
| Orange peel oil (92% limonene) encapsulated in amylose | Knowledge-driven AI | Fuzzy logic | Model limonene release from amylose nanostructures. | Fuzzy modelling accurately predicted release kinetics for GI-targeted delivery. | (Ganje *et al.*, 2019) |
| Coffee beverage infused with *Sargassum wightii* powder | Knowledge-driven AI | Fuzzy logic | Assess phytochemical and sensory changes in seaweed-enriched coffee. | 1% seaweed increased flavonoids and maintained consumer acceptability. | (Kumar *et al.*, 2019) |
| Lanmaoa asiatica, Butyriboletus roseoflavus, and Rugiboletus extremiorientalis (Edible wild boletes) | Deep learning | ResNet | Estimate total phenolic content and identify mushroom species using spectroscopy and DL. | AI-enabled spectral analysis provided accurate, non-invasive TPC estimation and species identification. | (Chen *et al.*, 2022) |
| (Morus alba L.) Mulberries | Deep learning | DNN | Optimize anthocyanin extraction using enzyme-ultrasound assistance and predict antioxidant activity. | DL accurately modelled extraction efficiency and antioxidant potential. | (C. Zhang *et al.*, 2025) |
| *Pericarpium Citri Reticulatae* (dried tangerine peel) | Hybrid and integrative approaches | CNN + PLS-DA, LS-SVM, RF | Classify tangerine peel samples by storage age and assess bioactive changes using THz-TDS and CNN. | CNN accurately predicted storage age and detected spectral differences in bioactive profiles. | (Y. Liu *et al.*, 2023) |
| Bioactive food peptides (e.g., from milk and sardine) | Deep learning | Multi-task GCN | Predict health benefits and mechanisms of action of food peptides using multi-task GCN. | AI model predicted multiple functional benefits, including antihypertensive and anticancer effects. | (Fukunaga *et al.*, 2020) |
| *Lycium barbarum* (Goji berries) | Deep learning | 1 DCNN, CAM, SAM, Multi-task CNN | Predict bioactive composition using hyperspectral imaging and multi-task CNN. | Imaging-based CNN accurately estimated bioactive levels, enabling non-destructive quality monitoring. | (Hu *et al.*, 2024) |
| *Pleurotus ostreatus* (Oyster mushroom) | Hybrid and integrative approaches | ANN, MOPSO | Optimize the extraction parameters of bioactive compounds using hybrid ANN-PSO modelling. | Coupled models improved yield and bioactivity, enhancing extraction efficiency. | (Gürgen & Sevindik, 2022) |
| *Petroselinum crispum* (Parsley juice) | Computational intelligence / optimization | PSO | Optimize thermosonication for preserving bioactive compounds in parsley juice. | Thermosonication preserved chlorophyll and vitamin C better than pasteurization; AI guided optimization. | (Dulger Altıner *et al.*, 2024) |
| *Citrus unshiu (*Citrus unshiu peels) | Computational intelligence / optimization | PSO | Optimize ultrasound-assisted extraction of phenolics and flavonoids. | AI-driven models improved extraction efficiency, revealing high bioactive recovery potential. | (Poyraz *et al.*, 2023) |
| *Cocos nucifera* L. (Green coconut shells) | Hybrid and integrative approaches | ANN, PSO | Optimize extraction conditions for phenolics and antioxidants using ANN and PSO. | Optimization enhanced yields and antioxidant activity; extract showed antimicrobial effects. | (Singh *et al.*, 2023) |
| *Eleusine coracana* (Finger millet) | Hybrid and integrative approaches | ANN, PSO | Improve bioactive and functional traits through ultrasound hydration and AI modelling. | Ultrasound reduced antinutrients, increased phenolics, and improved functional properties. | (Dubey & Tripathy, 2024) |
| (*Corylus avellana* L.) Hazelnut | Data-driven AI (classical ML) | PLS-DA, Supervised MLD | Enhance metabolomic quality prediction using data fusion of GC×GC and LC-HRMS. | AI revealed synergistic metabolic traits linked to quality parameters. | (Squara *et al.*, 2024) |
| Vegetables rich in flavonoids (USDA database) | Hybrid and integrative approaches | RF, SVM, MLP, K-NN, PSO + RST | Predict antioxidant capacity based on flavonoid descriptors using ML and DL models. | RF model showed highest accuracy; TPC was the most predictive variable. | (Guardado Yordi *et al.*, 2019) |
| Solanum lycopersicum (Tomato) | Deep learning | BPNN | Predict internal bioactive levels using multispectral imaging and BPNN. | multispectral imaging enabled accurate, non-invasive estimation of internal quality traits. | (C. Liu *et al.*, 2015) |
| *Juglans nigra* (black walnut), *Carthamus tinctoriusa* (fflower), *Zingiber officinale* (ginger), *Ficus carica* (Fig), *Zea mays* (corn), and *Piper nigrum* (pepper) | Deep Learning | DNN, RF | Identify food molecules interacting with Alzheimer’s disease targets using AI screening. | DL efficiently screened large compound libraries and predicted neuroprotective interactions. | (Zhou *et al.*, 2025) |
| Calf cruor (slaughterhouse by-product) | Data-driven AI (classical ML) | RF | Identify antimicrobial peptides from slaughterhouse by-products using ML-assisted screening. | ML predicted functional AMPs validated experimentally; enhanced biopreservation discovery. | (Sanchez-Reinoso *et al.*, 2025) |
| Whey protein hydrolysate | Deep learning | LLMs | Optimize enzymatic hydrolysis for antihypertensive peptide production using DL. | LLMs identified optimal enzymes and conditions, improving peptide bioactivity. | (Jiang *et al.*, 2025) |
| Baked goods from native grains | Data-driven AI (classical ML) | SVM | Assess effects of baking on antioxidant capacity in cereal-pulse flours. | AI accurately predicted total antioxidant capacity variations; baking influenced bioactivity depending on matrix. | (Rico *et al.*, 2023) |
| *Padina pavonica* (seaweed) | Data-driven AI (classical ML) | LR, SVM | Evaluate nanoformulated algae’s therapeutic effects on fatty liver using ML models. | LR and SVM models predicted efficacy; treatment improved liver markers significantly. | (Teng *et al.*, 2023) |
| Bee Pollen | Data-driven AI (classical ML) | RF | Predict crude protein content from digital images using RF. | RF model accurately estimated protein levels from image data. | (Breda *et al.*, 2024) |
| *Sus scrofa domesticus (*Phenolic antioxidants and Lard) | Deep learning | ELECTRA | Predict antioxidant interaction types (synergistic, additive, antagonistic) using DL. | Model accuracy improved with experimental data integration; structure alone was insufficient. | (Ayres *et al.*, 2023) |
| Mushroom-derived compounds (NA.) | Hybrid and integrative approaches | CP-ANN, SVM | Predict antioxidant potential using QSAR models and molecular descriptors. | CP-ANN and SVM models reliably estimated antioxidant activity. | (Martinčič *et al.*, 2015) |
| *Solanum tuberosum* (Potato powder) | Deep learning | ANN | Model acrylamide reduction by flavonoids using ANN and MLR. | Both models predicted acrylamide reduction effectively, offering low-cost assessment tools. | (Cheng *et al.*, 2015) |
| *Theobroma cacao* (Fermented cocoa beans) | Data-driven AI (classical ML) | PLS | Predict fermentation index, polyphenols, and antioxidants using hyperspectral imaging. | Hyperspectral imaging models accurately estimated internal quality traits non-destructively. | (Caporaso *et al.*, 2018) |
| Waste Oil (NA.) | Deep learning | ANN | Model fermentation for glyco-lipo-peptide production using ANN. | ANN predicted yield more accurately than statistical methods; improved optimization. | (Ekpenyong *et al.*, 2021) |
| *Cucurbita pepo* (Pumpkin seeds) | Deep learning | ANN | Optimize umami-related metabolite production using AI-guided enzymatic digestion. | AI strategy enhanced metabolite yield and diversity; supported flavor development. | (de la Hoz *et al.*, 2025) |
| *Ziziphus jujuba Mill.* (Jujube vinegar) | Deep learning | ANN | Compare ultrasound vs. thermal pasteurization on vinegar bioactives using ANN. | Ultrasound-preserved phenolics and volatiles; ANN-guided process optimization. | (Yıkmış *et al.*, 2022) |
| *Juglans regia* (Walnut) | Data-driven AI (classical ML) | RF | Identify anxiolytic peptides using multivariate analysis and RF. | RF predicted peptide activity effectively; promising candidates were identified. | (Hong *et al.*, 2025) |
| *Olea europaea* (Extra virgin olive oil) | Data-driven AI (classical ML) | K-NN | Assess bioactive compound content variation across olive oil samples. | Significant differences were linked to source and processing conditions. | (Różańska *et al.*, 2020) |
| ***Curcuma spp.*** ) Curcumae Radix( | Data-driven AI (classical ML) | K-NN, SVM, SVMR, BT, BTR, LDA | Discriminate species and predict compound content using spectrometry and chemometrics. | Combined models accurately classified species and predicted compound levels. | (Wang *et al.*, 2021) |
| *Helianthus annuus* L. (Sunflower microgreen) | Data-driven AI (classical ML) | K-NN, RR | Predict phenolic and flavonoid levels during growth using hyperspectral imaging. | hyperspectral imaging enabled non-invasive spatial and temporal monitoring of bioactive changes. | (Boonrat *et al.*, 2025) |
| *Vigna radiata* (Mung bean protein hydrolysate) | Deep learning | Bi-LSTM (AOPP) | Predict and analyze structure–activity of antioxidant peptides | AOPP accurately identified antioxidant peptides; validation confirmed key structural features linked to activity. | (Li *et al.*, 2025) |
| *Medicago truncatula* (root and leaves of legume) | Data-driven AI (classical ML) | K-means | Identify metabolite markers of abiotic and biotic stress | K-means clustering identified stress-responsive metabolites including flavonoids and isoflavonoids | (Dickinson *et al.*, 2018) |
| (*Prunus armeniaca* L.) Apricot kernels | Data-driven AI (classical ML) | RF + SHAP | Identify compounds contributing to antioxidant activity after fermentation | SHAP revealed hydroxybenzoic acid, L-cystine, and L-anserine as major contributors to antioxidant indices | (Zhao *et al.*, 2025) |

AOPP: Antioxidant Oligopeptide Predictor, ANN: Artificial Neural Network, BioDeepNat: Bioinformatics-integrated Deep Neural Analysis Tool, Bi-LSTM: bidirectional long-short-term memo, BPNN: Back Propagation Neural Network, BT: Bagged Trees, BTR: Bagged Trees Regression, CAM: Channel Attention Module, CNN: Convolutional Neural Network, CP-ANN: Counter-Propagation Artificial Neural Network, DNN: Deep Neural Networks, ELECTRA: Efficiently Learning an Encoder that Classifies Token Replacements Accurately, FL: Fuzzy Logic, GB: Gradient Boosting, GBM: Gradient Boosting Machine, GCN: Graph Convolutional Neural Network, GPR: Gaussian Process Regression, K-NN: K-Nearest Neighbor, LDA: Linear Discriminant Analysis, LLMs: Large Language Models, LR: Logistic Regression, LS-SVM: Least Squares Support Vector Machines, MLD: Mid-Level Data Fusion, MOPSO: Multiobjective Particle Swarm Optimization, PSO: Particle Swarm Optimization, PLS: Partial Least Squares, PLS-DA: Partial Least Squares-Discriminant Analysis, ResNet: Residual Convolutional Neural Network, RF: Random Forest, RR: Ridge Regression, SAM: Spectral Attention Module, SHAP: SHapley Additive exPlanations, SVM: Support Vector Machine, SVMR: Support Vector Machine Regression, XAI: Explainable Artificial Intelligence, 1D-CNN: One-Dimensional Convolutional Neural Network, Bidirectional Long Short-Term Memory, NA: unspecified species

| **Table A.2:** Selected studies employing artificial intelligence in personalized nutrition. | | | | | | |
| --- | --- | --- | --- | --- | --- | --- |
| **Food Targeted** | **AI Model / Platform Used** | **Personalization Basis** | **Objective** | **Personalization Outcome** | **Sample Size / Population** | **DOI** |
| Mediterranean and plant-based foods | PSO-SA integrated with AHP | Client-specific dietary needs, preferences, health goals | Generate personalized, nutritionally sound meal plans | High-adherence, tailored weekly meal plans for diabetics and elderly | 30 user profiles with diabetes, elderly included | (Sarani Rad *et al.*, 2024) |
| Customizable menus | Fuzzy logic for dynamic menu generation | Individual dietary needs and preferences | Design flexible nutritional menus based on environment and user profiles | Personalized adaptive menus | NA | (Karim El *et al.*, 2025) |
| Various global foods | FoodRecNet | User health, diet, religion, allergy, culture, demographics | Provide real-time personalized food recommendation | Effective preference-health balancing model | 3,335,492 records; 54,554 images | (Hamdollahi Oskouei & Hashemzadeh, 2023) |
| General meals (canteen-style, varied) | Smartphone app with personalized nutrition engine | User intake data, dietary goals, lifestyle | Evaluate effectiveness of AI-supported meal feedback on health outcomes | the study is ongoing and no results have been reported | Phase I: 170 randomized; Phase II: ~800 open users | (Feng *et al.*, 2023) |
| Items from Kaggle + NHANES datasets (e.g., spinach, kale) | Nutrient-similarity engine + rule-based health checker | Dietary intake, health risks (e.g., diabetes, obesity) | Recommend similar foods + evaluate diet-health compatibility | Suggested nutrient-aligned alternatives with >0.99 similarity | Dataset-based; 7,413 food records | (Olutunde *et al.*, 2024) |
| Tailored dietary plans (not item-specific) | Microbiome-based AI diet planning engine | Gut microbiota profile (IBS patients) | Compare the AI-personalized diet with standard low-FODMAP approach | Greater microbiome diversity and symptom reduction | 121 participants | (Tunali *et al.*, 2024) |
| Diverse food patterns (MFP data) | Many-objective optimization (SPEA2, NSGA-II, SPEA2+SDE) | User preferences, dietary patterns, nutrition values, and food diversity | Develop a personalized food recommender optimizing four health-related objectives | Balanced, health-aware, and preference-aligned recommendations | 1.9 million records, 71,000 food items, ~9,800 users | (J. Zhang *et al.*, 2022) |
| 12 diet types (e.g., DASH, (paleo, vegan diet) | Fuzzy AHP + Cuckoo Optimization + Fuzzy TOPSIS | Age, gender, height, weight, BP, heart rate | Rank diets based on health profile using hybrid MCDM | Custom diet ranking per individual profile | 20 participants | (Haseena *et al.*, 2022) |

AHP: Analytic Hierarchy Process, BP: Blood Pressure, CONOPT3: Constrained Optimization, version 3, DASH: Dietary Approaches to Stop Hypertension, FODMAP: Fermentable Oligosaccharides, Disaccharides, Monosaccharides, and Polyols, FoodRecNet: Food Recommendation Network ,GAMS: General Algebraic Modeling System, IBS: Irritable Bowel Syndrome, MCDM: Multi-Criteria Decision Making, MFP: MyFitnessPal, NHANES: National Health and Nutrition Examination Survey, NSGA-II: Non-dominated Sorting Genetic Algorithm II, PSO-SA: Particle Swarm Optimization–Simulated Annealing, SDE: Shift-based Density Estimation, , SPEA: Strength Pareto Evolutionary Algorithm, TOPSIS: Technique for Order of Preference by Similarity to Ideal Solution.

***References:***

Ayres, L., Benavidez, T., Varillas, A., Linton, J., Whitehead, D. C., & Garcia, C. D. (2023). Predicting Antioxidant Synergism via Artificial Intelligence and Benchtop Data. *Journal of Agricultural and Food Chemistry, 71*(42), 15644-15655. <https://doi.org/10.1021/acs.jafc.3c05462>

Basak, S. (2018). The use of fuzzy logic to determine the concentration of betel leaf essential oil and its potency as a juice preservative. *Food Chemistry, 240*, 1113-1120. <https://doi.org/10.1016/j.foodchem.2017.08.047>

Boonrat, P., Patel, M., Pengphorm, P., Detarun, P., & Daengngam, C. (2025). Hyperspectral Imaging for the Dynamic Mapping of Total Phenolic and Flavonoid Contents in Microgreens. *7*(4), 107. <https://doi.org/10.3390/agriengineering7040107>

Breda, L. S., de Melo Nascimento, J. E., Alves, V., de Alencar Arnaut de Toledo, V., de Lima, V. A., & Felsner, M. L. (2024). Green and fast prediction of crude protein contents in bee pollen based on digital images combined with Random Forest algorithm. *Food Res Int, 179*, 113958. <https://doi.org/10.1016/j.foodres.2024.113958>

Brillante, L., Gaiotti, F., Lovat, L., Vincenzi, S., Giacosa, S., Torchio, F., Segade, S. R., Rolle, L., & Tomasi, D. (2015). Investigating the use of gradient boosting machine, random forest and their ensemble to predict skin flavonoid content from berry physical–mechanical characteristics in wine grapes. *Computers and Electronics in Agriculture, 117*, 186-193. <https://doi.org/10.1016/j.compag.2015.07.017>

Caporaso, N., Whitworth, M. B., Fowler, M. S., & Fisk, I. D. (2018). Hyperspectral imaging for non-destructive prediction of fermentation index, polyphenol content and antioxidant activity in single cocoa beans. *Food Chem, 258*, 343-351. <https://doi.org/10.1016/j.foodchem.2018.03.039>

Chen, X., Liu, H., Li, J., & Wang, Y. (2022). Rapid identification of total phenolic content levels in boletes by two-dimensional correlation spectroscopy combined with deep learning. *Vibrational Spectroscopy, 121*, 103404. <https://doi.org/10.1016/j.vibspec.2022.103404>

Cheng, J., Chen, X., Zhao, S., & Zhang, Y. (2015). Antioxidant-capacity-based models for the prediction of acrylamide reduction by flavonoids. *Food Chemistry, 168*, 90-99. <https://doi.org/10.1016/j.foodchem.2014.07.008>

de la Hoz, A., Caripán, P., Moltedo, B., Ferrada, N., & Contreras, R. A. (2025). A metabolomic approach of AI-driven enzymatic digestion of pumpkin seed flour for producing umami metabolites. *International Journal of Gastronomy and Food Science, 39*, 101117. <https://doi.org/10.1016/j.ijgfs.2025.101117>

Dickinson, E., Rusilowicz, M. J., Dickinson, M., Charlton, A. J., Bechtold, U., Mullineaux, P. M., & Wilson, J. (2018). Integrating transcriptomic techniques and k-means clustering in metabolomics to identify markers of abiotic and biotic stress in Medicago truncatula. *Metabolomics, 14*(10), 126. <https://doi.org/10.1007/s11306-018-1424-y>

Dubey, A., & Tripathy, P. P. (2024). Ultrasound-mediated hydration of finger millet: Effects on antinutrients, techno-functional and bioactive properties, with evaluation of ANN-PSO and RSM optimization methods. *Food Chem, 435*, 137516. <https://doi.org/10.1016/j.foodchem.2023.137516>

Dulger Altıner, D., Yıkmış, S., Şimşek, M. A., Türkol, M., Tokatlı Demirok, N., & Celik, G. (2024). Impact of Thermosonication Treatment on Parsley Juice: Particle Swarm Algorithm (PSO), Multiple Linear Regression (MLR), and Response Surface Methodology (RSM). *ACS Omega, 9*(27), 29585-29597. <https://doi.org/10.1021/acsomega.4c02749>

Ekpenyong, M., Asitok, A., Antai, S., Ekpo, B., Antigha, R., & Ogarekpe, N. (2021). Statistical and Artificial Neural Network Approaches to Modeling and Optimization of Fermentation Conditions for Production of a Surface/Bioactive Glyco-lipo-peptide. *International Journal of Peptide Research and Therapeutics, 27*(1), 475-495. <https://doi.org/10.1007/s10989-020-10094-8>

Feng, J., Liu, H., Mai, S., Su, J., Sun, J., Zhou, J., Zhang, Y., Wang, Y., Wu, F., Zheng, G., & Zhu, Z. (2023). Protocol of a parallel, randomized controlled trial on the effects of a novel personalized nutrition approach by artificial intelligence in real world scenario. *BMC Public Health, 23*(1), 1700. <https://doi.org/10.1186/s12889-023-16434-9>

Fukunaga, I., Sawada, R., Shibata, T., Kaitoh, K., Sakai, Y., & Yamanishi, Y. (2020). Prediction of the Health Effects of Food Peptides and Elucidation of the Mode-of-action Using Multi-task Graph Convolutional Neural Network. *39*(1-2), 1900134. <https://doi.org/10.1002/minf.201900134>

Ganje, M., Jafari, S. M., Tamadon, A. M., Niakosari, M., & Maghsoudlou, Y. (2019). Mathematical and fuzzy modeling of limonene release from amylose nanostructures and evaluation of its release kinetics. *Food Hydrocolloids, 95*, 186-194. <https://doi.org/10.1016/j.foodhyd.2019.04.045>

Gu, M., Li, C., Chen, L., Li, S., Xiao, N., Zhang, D., & Zheng, X. (2023). Insight from untargeted metabolomics: Revealing the potential marker compounds changes in refrigerated pork based on random forests machine learning algorithm. *Food Chem, 424*, 136341. <https://doi.org/10.1016/j.foodchem.2023.136341>

Guardado Yordi, E., Koelig, R., Matos, M. J., Pérez Martínez, A., Caballero, Y., Santana, L., Pérez Quintana, M., Molina, E., & Uriarte, E. (2019). Artificial Intelligence Applied to Flavonoid Data in Food Matrices. *Foods, 8*(11), 573. <https://doi.org/10.3390/foods8110573>

Guiné, R. P. F., Barroca, M. J., Gonçalves, F. J., Alves, M., Oliveira, S., & Mendes, M. (2015). Artificial neural network modelling of the antioxidant activity and phenolic compounds of bananas submitted to different drying treatments. *Food Chemistry, 168*, 454-459. <https://doi.org/10.1016/j.foodchem.2014.07.094>

Gürgen, A., & Sevindik, M. (2022). Application of artificial neural network coupling multiobjective particle swarm optimization algorithm to optimize Pleurotus ostreatus extraction parameters. *46*(11), e16949. <https://doi.org/10.1111/jfpp.16949>

Hamdollahi Oskouei, S., & Hashemzadeh, M. (2023). FoodRecNet: a comprehensively personalized food recommender system using deep neural networks. *Knowledge and Information Systems, 65*(9), 3753-3775. <https://doi.org/10.1007/s10115-023-01897-4>

Haseena, S., Saroja, S., & Revathi, T. (2022). A fuzzy approach for multi criteria decision making in diet plan ranking system using cuckoo optimization. *Neural Computing and Applications, 34*(16), 13625-13638. <https://doi.org/10.1007/s00521-022-07163-y>

Hong, Z., Cheng, Y., Zhou, M., Wang, S., Li, X., Feng, S., Su, G., Zheng, L., & Zhao, M. (2025). Exploring anxiolytic peptides derived from walnut protein by an integrated approach of multivariate analysis, random forest methodology. *Food Bioscience, 66*, 106271. <https://doi.org/10.1016/j.fbio.2025.106271>

Hu, H., Mei, Y., Wei, Y., Xu, Z., Zhao, Y., Xu, H., Mao, X., & Huang, L. (2024). Chemical composition prediction in goji (Lycium barbarum) using hyperspectral imaging and multi-task 1DCNN with attention mechanism. *LWT, 204*, 116436. <https://doi.org/10.1016/j.lwt.2024.116436>

Jandoust, S., Shojaeiyan, A., Ayyari, M., Tohidfar, M., Ahmadi, H., & Ebrahimi, S. N. (2023). Using artificial neural network for prediction of accumulation of bioactive compounds in Momordica charantia through application of different elicitors. *Industrial Crops and Products, 192*, 115984. <https://doi.org/10.1016/j.indcrop.2022.115984>

Jiang, S., Mo, F., Li, W., Yang, S., Li, C., & Jiang, L. (2025). Deep Learning-Driven Optimization of Antihypertensive Properties from Whey Protein Hydrolysates: A Multienzyme Approach. *J Agric Food Chem, 73*(2), 1373-1388. <https://doi.org/10.1021/acs.jafc.4c10830>

Karim El, M., Abdellah, A., Fatima, B., Aayah, H., Alina-Mihaela, P., Saliha, C., & Hicham, B. (2025). Fuzzy Modeling to Personalized Nutritional Menu. *Current Nutrition & Food Science, 21*(1), 55-66. <https://doi.org/10.2174/0115734013293555240319070046>

Kongsompong, S., E-kobon, T., & Chumnanpuen, P. (2021). K-Nearest Neighbor and Random Forest-Based Prediction of Putative Tyrosinase Inhibitory Peptides of Abalone Haliotis diversicolor. *Molecules, 26*(12), 3671. <https://doi.org/10.3390/molecules26123671>

Kumar, Y., Tarafdar, A., Kumar, D., & Badgujar, P. C. (2019). Effect of Indian brown seaweed Sargassum wightii as a functional ingredient on the phytochemical content and antioxidant activity of coffee beverage. *Journal of Food Science and Technology, 56*(10), 4516-4525. <https://doi.org/10.1007/s13197-019-03943-y>

Lee, S. Y., Mediani, A., Maulidiani, M., Khatib, A., Ismail, I. S., Zawawi, N., & Abas, F. (2018). Comparison of partial least squares and random forests for evaluating relationship between phenolics and bioactivities of Neptunia oleracea. *98*(1), 240-252. <https://doi.org/https://doi.org/10.1002/jsfa.8462>

Li, W., Liu, X., Liu, Y., & Zheng, Z. (2025). High-Accuracy Identification and Structure-Activity Analysis of Antioxidant Peptides via Deep Learning and Quantum Chemistry. *J Chem Inf Model, 65*(2), 603-612. <https://doi.org/10.1021/acs.jcim.4c01713>

Liu, C., Liu, W., Chen, W., Yang, J., & Zheng, L. (2015). Feasibility in multispectral imaging for predicting the content of bioactive compounds in intact tomato fruit. *Food Chemistry, 173*, 482-488. <https://doi.org/10.1016/j.foodchem.2014.10.052>

Liu, Y., Pu, H., Li, Q., & Sun, D. W. (2023). Discrimination of Pericarpium Citri Reticulatae in different years using Terahertz Time-Domain spectroscopy combined with convolutional neural network. *Spectrochim Acta A Mol Biomol Spectrosc, 286*, 122035. <https://doi.org/10.1016/j.saa.2022.122035>

Luka, B. S., Yuguda, T. K., Adnouni, M., Zakka, R., Abdulhamid, I. B., & Gargea, B. G. (2022). Drying temperature-dependent profile of bioactive compounds and prediction of antioxidant capacity of cashew apple pomace using coupled Gaussian Process Regression and Support Vector Regression (GPR-SVR) model. *Heliyon, 8*(9), e10461. <https://doi.org/10.1016/j.heliyon.2022.e10461>

Ma, J., Zhou, X., Xie, B., Wang, C., Chen, J., Zhu, Y., Wang, H., Ge, F., & Huang, F. (2023). Application for Identifying the Origin and Predicting the Physiologically Active Ingredient Contents of Gastrodia elata Blume Using Visible–Near-Infrared Spectroscopy Combined with Machine Learning. *Foods, 12*(22), 4061. <https://doi.org/10.3390/foods12224061>

Martinčič, R., Kuzmanovski, I., Wagner, A., & Novič, M. (2015). Development of models for prediction of the antioxidant activity of derivatives of natural compounds. *Analytica Chimica Acta, 868*, 23-35. <https://doi.org/10.1016/j.aca.2015.01.050>

Mishra, D. S., Berwal, M. K., Singh, A., Singh, A. K., Rao, V. V. A., Yadav, V., & Sharma, B. D. (2022). Phenotypic diversity for fruit quality traits and bioactive compounds in red-fleshed guava: Insights from multivariate analyses and machine learning algorithms. *South African Journal of Botany, 149*, 591-603. <https://doi.org/10.1016/j.sajb.2022.06.043>

Olutunde, T., Ani, C. L., & Adesue, G. A. (2024). Leveraging Machine Learning for Personalized Dietary Recommendations, Nutritional Patterns, and Health Outcome Predictions. *Journal of Science Research and Reviews, 1*(2), 43-56. <https://doi.org/10.70882/josrar.2024.v1i2.40>

Pérez-Rodríguez, M., Gaiad, J. E., Hidalgo, M. J., Avanza, M. V., & Pellerano, R. G. (2019). Classification of cowpea beans using multielemental fingerprinting combined with supervised learning. *Food Control, 95*, 232-241. <https://doi.org/10.1016/j.foodcont.2018.08.001>

Poyraz, Ç., Küçükyıldız, G., Kırbaşlar, Ş. İ., Ciğeroğlu, Z., & Şahin, S. (2023). Valorization of Citrus unshiu biowastes to value-added products: an optimization of ultrasound-assisted extraction method using response surface methodology and particle swarm optimization. *Biomass Conversion and Biorefinery, 13*(5), 3719-3729. <https://doi.org/10.1007/s13399-021-01329-9>

Rebollo-Hernanz, M., Cañas, S., Taladrid, D., Segovia, Á., Bartolomé, B., Aguilera, Y., & Martín-Cabrejas, M. A. (2021). Extraction of phenolic compounds from cocoa shell: Modeling using response surface methodology and artificial neural networks. *Separation and Purification Technology, 270*, 118779. <https://doi.org/10.1016/j.seppur.2021.118779>

Rico, D., Cano, A. B., Alvarez Alvarez, S., Rio Briones, G., & Martin Diana, A. B. (2023). Study of the Total Antioxidant Capacity (TAC) in Native Cereal-Pulse Flours and the Influence of the Baking Process on TAC Using a Combined Bayesian and Support Vector Machine Modeling Approach. *Foods, 12*(17), 3208. <https://doi.org/10.3390/foods12173208>

Różańska, A., Russo, M., Cacciola, F., Salafia, F., Polkowska, Ż., Dugo, P., & Mondello, L. (2020). Concentration of Potentially Bioactive Compounds in Italian Extra Virgin Olive Oils from Various Sources by Using LC-MS and Multivariate Data Analysis. *Foods, 9*(8), 1120. <https://doi.org/10.3390/foods9081120>

Sampaio, P. N., & Calado, C. C. R. (2024). Enhancing Bioactive Compound Classification through the Synergy of Fourier-Transform Infrared Spectroscopy and Advanced Machine Learning Methods. *Antibiotics, 13*(5), 428. <https://doi.org/10.3390/antibiotics13050428>

Sanchez-Reinoso, Z., García-Vela, S., Clément, J.-P., & Bazinet, L. (2025). Combining statistical, machine learning and experimental approaches for screening of novel antimicrobial peptides of calf cruor hydrolysates. *Food Bioscience, 65*, 105734. <https://doi.org/10.1016/j.fbio.2024.105734>

Sarani Rad, F., Amiri, M., & Li, J. (2024). Optimizing Nutritional Decisions: A Particle Swarm Optimization-Simulated Annealing-Enhanced Analytic Hierarchy Process Approach for Personalized Meal Planning. *Nutrients, 16*(18), 3117. <https://doi.org/10.3390/nu16183117>

Sharifi, A., & Khoshnoudi-Nia, S. (2022). Ranking novel extraction systems of seedless barberry (Berberis Vulgaris) bioactive compounds with fuzzy logic-based term weighting scheme. *Sustainable Chemistry and Pharmacy, 25*, 100561. <https://doi.org/10.1016/j.scp.2021.100561>

Singh, P., Pandey, V. K., Chakraborty, S., Dash, K. K., Singh, R., Mukarram Shaikh, A., & Béla, K. (2023). Ultrasound-assisted extraction of phytochemicals from green coconut shell: Optimization by integrated artificial neural network and particle swarm technique. *Heliyon, 9*(12), e22438. <https://doi.org/10.1016/j.heliyon.2023.e22438>

Squara, S., Caratti, A., Fina, A., Liberto, E., Koljancic, N., Spanik, I., Genova, G., Castello, G., Bicchi, C., de Villiers, A., & Cordero, C. (2024). Artificial intelligence decision making tools in food metabolomics: Data fusion unravels synergies within the hazelnut (Corylus avellana L.) metabolome and improves quality prediction. *Food Res Int, 194*, 114873. <https://doi.org/10.1016/j.foodres.2024.114873>

Taherkhani, A., Sharifi, A., & Koubaa, M. (2024). Optimization of Bioactive Compound Extraction from Iranian Brown Macroalgae Nizimuddinia zanardini with Ultrasound and Microwave Methods Using Fuzzy Logic. *13*(23), 3837.

Teng, S., Zheng, N., Al-Huqail, A. A., Lu, Y., Ali, E., Ali, H. E., & Zhao, H. (2023). Effect of nanoparticle macroalgae in the treatment of fatty liver disease using logistic regression, and support vector machine. *Environmental Research, 224*, 115426. <https://doi.org/10.1016/j.envres.2023.115426>

Tian, Y., Zhang, Z., & Yan, A. (2023). Discovering the Active Ingredients of Medicine and Food Homologous Substances for Inhibiting the Cyclooxygenase-2 Metabolic Pathway by Machine Learning Algorithms. *Molecules, 28*(19), 6782. <https://doi.org/10.3390/molecules28196782>

Tunali, V., Arslan, N. C., Ermis, B. H., Dervis Hakim, G., Gundogdu, A., Hora, M., & Nalbantoglu, O. U. (2024). A Multicenter Randomized Controlled Trial of Microbiome-Based Artificial Intelligence-Assisted Personalized Diet vs Low-Fermentable Oligosaccharides, Disaccharides, Monosaccharides, and Polyols Diet: A Novel Approach for the Management of Irritable Bowel Syndrome. *Am J Gastroenterol, 119*(9), 1901-1912. <https://doi.org/10.14309/ajg.0000000000002862>

Wang, L., Ren, X., Wang, Y., Liu, X., Dong, Y., Ma, J., Song, R., Yu, A., Wei, J., Fan, Q., Wang, X., & She, G. (2021). HPLC fingerprint and UV–Vis spectroscopy coupled with chemometrics for Curcumae radix species discrimination and three bioactive compounds prediction. *Microchemical Journal, 166*, 106254. <https://doi.org/10.1016/j.microc.2021.106254>

Yıkmış, S., Altıner, D. D., Ozer, H., Levent, O., Celik, G., & Çöl, B. G. (2022). Modeling and optimization of bioactive compounds from jujube (Ziziphus jujuba Mill.) vinegar using response surface methodology and artificial neural network: Comparison of ultrasound processing and thermal pasteurization. *46*(11), e17102. <https://doi.org/https://doi.org/10.1111/jfpp.17102>

Younis, K., Ahmad, S., Osama, K., & Malik, M. A. (2019). Optimization of de-bittering process of mosambi (Citrus limetta) peel: Artificial neural network, Gaussian process regression and support vector machine modeling approach. *42*(6), e13185. <https://doi.org/https://doi.org/10.1111/jfpe.13185>

Zhang, C., Ding, W., Mamattursun, A., Ma, X., Qi, S., Wu, Y., Zhang, J., & Ma, X. (2025). Optimization of enzyme-ultrasound assisted extraction from mulberries anthocyanins based on response surface methodology and deep neural networks and analysis of in vitro antioxidant activities. *Food Chemistry, 478*, 143597. <https://doi.org/10.1016/j.foodchem.2025.143597>

Zhang, J., Li, M., Liu, W., Lauria, S., & Liu, X. (2022). Many-objective optimization meets recommendation systems: A food recommendation scenario. *Neurocomputing, 503*, 109-117. <https://doi.org/10.1016/j.neucom.2022.06.081>

Zhao, Z., Kantono, K., Kam, R., Le, T. T., Kitundu, E., Chen, T., & Hamid, N. (2025). Improving the Bioactivities of Apricot Kernels Through Fermentation: Investigating the Relationship Between Bioactivities, Polyphenols, and Amino Acids Through the Random Forest Regression XAI Approach. *14*(5), 845. <https://doi.org/10.3390/foods14050845>

Zhou, J., Li, C., Kim, Y. K., & Park, S. (2025). Bioinformatics and Deep Learning Approach to Discover Food-Derived Active Ingredients for Alzheimer’s Disease Therapy. *14*(1), 127.

Zhou, J., Yuan, H., & Park, S. (2024). Predicting structure-targeted food bioactive compounds for middle-aged and elderly Asians with myocardial infarction: insights from genetic variations and bioinformatics-integrated deep learning analysis. *Food & Function, 15*(18), 9497-9511. <https://doi.org/10.1039/D4FO00591K>
